# Mathematical modeling of the cortisol stress response to develop indicators that are applicable across studies

**DOI:** 10.1016/j.ynstr.2026.100790

**Published:** 2026-03-06

**Authors:** Laura de Nooij, Jonathan F. Posthuma, Robert Miller, Milou S.C. Sep, Conny Quaedflieg, Dennis Hernaus, STRESS-EU Consortium, Christiaan H. Vinkers, Erno J. Hermans

**Affiliations:** a Radboud University Medical Centre, Donders Institute for Brain, Cognition and Behaviour, the Netherlands; b Radboud University, Donders Institute for Brain, Cognition and Behaviour, the Netherlands; c Psychologische Hochschule Berlin, Germany; d Department of Psychiatry, Amsterdam University Medical Center Location Vrije Universiteit Amsterdam, the Netherlands; e GGZ inGeest Mental Health Care, Amsterdam, the Netherlands; f Amsterdam Neuroscience, Mood, Anxiety, Psychosis, Sleep & Stress Program, Amsterdam, the Netherlands; g Amsterdam Public Health, Mental Health Program, Amsterdam, the Netherlands; h Department of Neuropsychology and Psychopharmacology, Faculty of Psychology and Neuroscience, Maastricht University, Maastricht, the Netherlands; i Department of Psychiatry & Neuropsychology, School for Mental Health and Neuroscience, Maastricht University, Maastricht, the Netherlands

**Keywords:** Salivary cortisol, Cortisol response, Area under the curve, Acute stress test, Stress induction, Statistical modeling

## Abstract

Growing interest in interindividual differences in stress neuroendocrinology has created a need to combine data from multiple laboratory acute stress induction studies to allow large-scale individual participant data (IPD) meta-analyses. However, established cortisol stress response indicators such as area under the curve (AUC) are inherently affected by sampling timing and duration, although to what extent remains unknown. Here, we leveraged a large, combined dataset (STRESS-EU; n = 1295) to develop novel model-based indicators that can accommodate variability in sampling schedules. These were based on modeled individual response curves that achieve full data inter- and extrapolation. We validated this method with simulated and independent data. Crucially, combined data simulations particularly showed higher accuracy for model-based versus conventional ‘observation-based’ AUC indicators with variability in sampling duration. In conclusion, our novel method harmonizes cortisol response indicator estimates for combined data, yielding opportunities for IPD meta-analyses of acute stress test studies that could greatly advance the field.

## 1 Introduction

Acute stressors trigger the release of cortisol from the adrenal glands as part of a major neuroendocrine regulatory system known as the hypothalamic-pituitary adrenal (HPA) axis (Tsigos and Chrousos, 2002). A commonly used approach to quantify HPA-axis activation is the measurement of cortisol release in response to laboratory acute stress tests that provide standardized stress induction procedures (Bali and Jaggi, 2015). Acute stress test studies have generated important insights, for example into HPA-axis dysregulation associated with clinical disorders such as depression and schizophrenia (Ciufolini et al., 2014; Zorn et al., 2017). Yet, many questions remain, for instance with regards to psychiatric comorbidity. The need to enhance power for analyses is illustrated by heterogeneous results across acute stress test studies that are due to the variability in acute stress test procedures and large individual differences in the cortisol stress response. The STRESS-EU database (Sep et al., 2024), which contains data from a growing number of individual studies employing acute stress tests with salivary cortisol measurements, has been established to address this problem. Combining data from studies with modest sample sizes together creates a valuable resource for individual participant data (IPD) meta-analyses, but is challenged by vast differences in timing and frequency of saliva sampling (‘sampling schedules’) that may bias commonly used metrics of the cortisol stress response.

Laboratory acute stress tests aim to induce a stress response via uncontrollability, unpredictability and/or social-evaluative threat (Dickerson and Kemeny, 2004). A challenge for data harmonization and cross-study comparability is that the exact procedures of well-validated protocols differ widely. For instance, the frequently-used Trier Social Stress Test (TSST) involves negative evaluation by social-non-responsive judges during a mock job interview presentation, in combination with a difficult mental arithmetic task (Kirschbaum et al., 1993; Kudielka et al., 2007). This procedure has also been adapted to a version for groups (gTSST) (von Dawans et al., 2011). Another commonly applied procedure is the socially evaluated cold pressor test (SECPT), in which participants are socially evaluated while immersing their hand in ice-cold water (Schwabe et al., 2008; Schwabe and Schächinger, 2018). The Maastricht Acute Stress Test (MAST) combines negative social evaluation, cold water, and arithmetic components from SECPT and TSST, and its imaging variant – the iMAST – replaces cold water with thermal stimulation to allow for functional magnetic resonance imaging (fMRI) compatibility (Quaedflieg et al., 2013; Smeets et al., 2012). Laboratory acute stress tests typically include repeated sampling of saliva for cortisol analyses, but with great variety in sampling schedules across studies. A golden standard for quantifying cortisol stress responses in combined datasets that consist of original laboratory acute stress studies with varying sampling schedules currently does not exist.

Established metrics to quantify cortisol stress response (‘cortisol summary indicators’) suffer from limitations that may exacerbate with combined data that contains variation in sampling schedules. Area under curve (AUC) measures are comprehensive measures that express total turnover of free (protein-unbound) salivary cortisol concentrations (AUC with respect to ground, AUCg) or change in cortisol turnover (AUC with respect to increase, AUCi) (Pruessner et al., 2003). These summary indicators aggregate repeated measurements over time to a single indicator with the aim of enhancing statistical power, although at the expense of information on temporal dynamics (Fekedulegn et al., 2007). By nature, AUC indicators are directly affected by sampling duration. This creates a bias when combining data from studies with different sampling schedules. Reactivity is another commonly used cortisol summary indicator. It reflects HPA-axis reactivity by subtraction of the cortisol value from prior to stress induction (‘baseline value’) from the cortisol value at the expected peak timing (Khoury et al., 2015). Maximum increase is an indicator that reflects the dynamic range of the system (Miller et al., 2018). The accuracy of reactivity and maximum increase indicators likely depends on the extent to which sampling times match peak timing; both sampling times and peak timing may differ between studies due to methodological differences. Although it is likely that common summary indicators of the stress response are affected by variability in sampling schedules, it remains unknown to what extent this variability poses a threat to the analysis of combined datasets and how this challenge should be addressed.

Here we present a novel method based on mathematical modeling that aims to address the shortcomings of cortisol summary indicators, particularly in the context of combining data with variability in sampling schedules for IPD meta-analyses. The method is developed and validated in three steps. In the first step, the STRESS-EU database of acute stress test studies is used to develop two different models of the cortisol stress response. Both models are created to inter- and extrapolate datapoints for individual participant data from acute stress test studies, so that indicators of the cortisol stress response can be extracted in a model-based manner. The amplitude scaling model assumes a uniform shape of the cortisol stress curve, whereas the multilevel model flexibly models the shape of individual cortisol response curves, while taking into account covariates such as the type of acute stress test and gender. Since any model represents a simplification of the real world, we did not have an a priori hypothesis on which of the two models would provide the best solution. In the second step, we evaluate our novel method with simulated data. After quantifying shortcomings of conventional observation-based summary indicators, we test whether model-based summary indicators would provide solutions to these shortcomings. Both models are therefore applied to high temporal-resolution simulated data that were previously (independently) generated based on realistic pharmacokinetic models. These generated data are downsampled to simulate different sampling schedules, but still show the advantage of underlying high temporal-resolution data that can provide “true” indicator values. To achieve validation of our method, model-based indicators are compared to conventional observation-based indicators in the light of “true” indicator values. In the third step, we apply our novel method to two acute stress test datasets for further validation and to illustrate derivation of model-based indicator values from real data.

## 2 Methods

### 2.1 Step 1: method development with STRESS-EU data

**Fig. 1:**
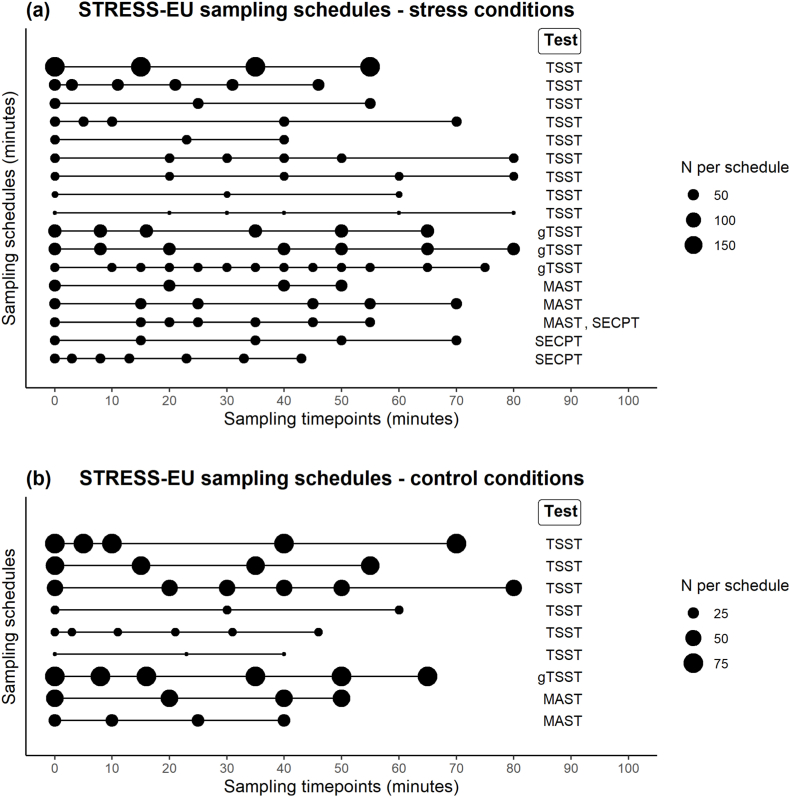
Sampling schedules (relative to stressor onset) within the final STRESS-EU database sample of the current study, for (a) stress conditions and (b) control conditions (only displayed for n ≥ 5).

We developed our mathematical models using data from the STRESS-EU database, a repository containing cortisol measurements from laboratory acute stress test studies (Bonapersona et al., 2022; Sep et al., 2024). In April 2023, all available STRESS-EU data from individuals aged 18-64 years old (*N* = 5102) were obtained. Firstly, timepoints of salivary cortisol measurements were referenced to the onset of the stress procedure. All observations from t = −20 until t = 2 min were considered a baseline measurement and set to t = 0 min. In case of multiple baseline measurements, these were averaged for a corrected baseline value at t = 0 min. No availability of a corrected t = 0 min baseline measurement resulted in participant exclusions (n = 1282). Participants with any reported (history of) psychiatric or otherwise potentially HPA-related diagnosis (n = 262) and data not collected in the afternoon (n = 946) were also excluded. Furthermore, the current study only considered studies that applied one of the following types of acute stress tests: TSST (Kirschbaum et al., 1993; Kudielka et al., 2007; stressor exposure time of 13 min including 3 min of anticipation), TSST for groups [gTSST] (von Dawans et al., 2011; stressor exposure time of 20 min excluding 10 min of preparation), (Socially Evaluated) Cold Pressor Test [(SE)CPT] (Hines and Brown, 1932; Schwabe et al., 2008; Schwabe and Schächinger, 2018, stressor exposure time of 3 min), (imaging) Maastricht Acute Stress Test [(i)MAST] (Quaedflieg et al., 2013; Smeets et al., 2012; stressor exposure time of 15 min including 5 min of preparation), or a no-stress control condition procedure of one of these tests (Wiemers et al., 2013); data from cumulative stress procedures (n = 594) and an aversive movie paradigm (n = 300) were excluded. Limiting the minimum sampling duration to 40 min after stressor onset did not result in any additional exclusions. Observations (samples) were excluded when they were sampled outside of the range from corrected baseline t = 0 to t = 80 min (750 observations) or when the cortisol value was classified as ±5 SD outlier (20 observations). The final dataset included n = 1295 participants (473 F, age *M* (*SD*) = 24.0 (8.0) years) from 31 studies with a total of 6735 salivary cortisol measurements (per participant: *M* (*SD*) = 5.2 (1.7), range 2-13). The different sampling schedules and acute stress tests within the STRESS-EU database of the current study are presented in Fig. 1.

All STRESS-EU data were partitioned into 80%-training and 20%-test datasets stratified based on study-identifier with python package scikit-learn (version 1.0.1) (Pedregosa et al., 2011). Here, we verified that they showed the expected distributions for stress versus control procedure (χ2 = 0.05,*p* = .81) and gender (χ2 = 0.29,*p* = .58). In the current study, training data was used for model selection and an initial model fit, which was then applied to test data to preclude model overfitting. Subsequently, final models were trained with the entire STRESS-EU dataset (training + test partition) to optimize their accuracy. Further evaluation of these models was independent of the Stress-EU dataset (i.e., models were applied to simulated and external data).

#### 2.1.1 Model origin and parameters

We applied two different modeling approaches: (1) a multilevel model and (2) an amplitude scaling model. These were applied to STRESS-EU data to estimate the (average) cortisol response curve. The kernel of both nonlinear models is formed by the density function of the gamma distribution. The choice for the gamma distribution is in line with and detailed by previous work (Miller et al., 2018; see pp. 198-199 and formulae 9-10). The gamma distribution is shaped by the parameters α and β (formula 1).

(1) $$f\left(x\right)=\frac{\beta^{\alpha }}{\Gamma \left(\alpha \right)}x^{\alpha -1}e^{-\beta x}$$

in this study, both cortisol stress response models predict the cortisol value for a given timepoint *t* (relative to stressor onset) based on a gamma distribution that is extended with two additional parameters *A* and *b*0 (formula 2).

(2) g(t)=A·f(t)+b0

Here, gamma distribution *f*(t) is multiplied by parameter *A* to scale the magnitude of the cortisol stress response. Baseline parameter *b*0 shifts the baseline from zero. This formula can be further extended by addition of two parameters *dT* and *b*1 (formula 3).

(3) g(t)=A·f(t−dT)+b0+b1(t−dT)

Parameter *dT* shifts timepoints and thus the peak of the response. Parameter *b*1 allows a “slow drift” in the cortisol stress response corresponding to the cortisol diurnal rhythm (Upton et al., 2023). The use of these additional two parameters was evaluated as part of the model selection procedure as described below.

#### 2.1.2 Model development and parameter selection for the multilevel model

The multilevel model assumes heterogeneity in the cortisol stress response. It allows variability in the shape of the response across individuals. This is achieved by incorporating relevant covariates and estimating individual-specific parameters while simultaneously modelling population-level effects. A key feature of the multilevel framework is *shrinkage*: individual parameter estimates are partially pooled toward the population mean, with the degree of shrinkage increasing as uncertainty increases (e.g., when fewer data points are available for an individual). The multilevel model was implemented in R version 4.3.1 with package saemix version 3.2 (Comets et al., 2017). Distribution families and initial parameter values were determined prior to the model selection procedure so that they resembled the expected shape of the cortisol stress response (Fig. S1 in **Supplementary Methods**). The model selection procedure that was applied to the training dataset consisted of three steps. Firstly, the impact of including parameters *dT* and *b*1 on the optimal trade-off between model fit and model complexity was evaluated using the Bayesian Information criterion (BIC) metric. This resulted in a final multilevel model with six parameters according to formula (3). Secondly, covariates condition (stress versus control), gender, age, and type of acute stress test were sequentially added and evaluated to optimize the model further. Here, the covariation for type of acute stress tests was implemented as four dummy coded variables for (1) all control conditions, (2) gTSST-stress (3) SECPT-stress and (4) MAST-stress; this left TSST-stress as reference category. Covariate effects on different parameters were only retained in case of an associated *p*-value below 5%, when they could be interpreted reliably (coefficient of variation ≤25%), and when it did not negatively affect BIC. The following covariate effects were included: gender for parameters *A* and *b*0; laboratory acute stress test dummy coded variable 1 (control conditions) for parameters *A*, *b*0, *b*1 and *dT*; and laboratory acute stress test dummy coded variable 3 (MAST) for parameters β and *b*0. Thirdly, heteroscedasticity was assessed by visual inspection, which resulted in specification of a “constant” error model. More details and code are available in Supplementary Methods 1.

#### 2.1.3 Model development and parameter selection for the amplitude scaling model

The amplitude scaling model assumes a cortisol stress response with similar shape across participants and only estimates individual response amplitudes. For this model, we only included subsets of data from stress condition participants that were classified as responders. This decision was based on the assumption that modeling of the average cortisol stress response shape would be most successful when based only on individuals that would show a cortisol stress response; and that this would hold even if it is subsequently also applied to control conditions or non-responder data (the expected amplitude would then be zero). Responders were classified according to an increase of 1.5 nmol/L from corrected baseline cortisol value to the maximum cortisol value from 15 to 45 min post stressor onset (Miller et al., 2013). After exclusion of individuals in control conditions and stress non-responders, the dataset for the amplitude scaling model included n = 507 participants (147 F, age M (*SD*) = 24.6 (9.1)) from 28 studies with a total of 2699 salivary cortisol measurements.

The amplitude scaling model was implemented in python using package lmfit version 1.2.1 (Newville et al., 2014). First, parameters were estimated from the training data to derive the average cortisol stress response of the training sample. Here, *b*0 was not freely estimated but fixed to the mean cortisol value of the training sample at baseline (t = 0 min). To select the best model, inclusion of parameters *dT* and *b*1 were evaluated according to Bayesian Information criterion (BIC). The optimal model for the average cortisol stress response did not contain these additional parameters; only the four parameters specified in formula (2). With the amplitude scaling model, the amplitude of the average cortisol stress response curve was re-estimated for each individual. Specifically, individual predictions were achieved for each participant with the following three steps: (1) all cortisol values were normalized by subtracting the participant's observed cortisol value at baseline (t = 0 min), (2) these normalized data were used to scale the amplitude of the average cortisol response curve, and (3) the baseline of the cortisol response curve was shifted by adding the participant's observed baseline value.

#### 2.1.4 Calculation of model-based and conventional cortisol summary indicators

Application of either model allows subsequent derivation of model-based indicators of the cortisol stress response. These model-based indicators are calculated based on model predictions that inter- and extrapolate all datapoints, instead of the actual observations. In the current study, we investigated novel model-based indicators of AUCi, AUCg, reactivity and maximum increase and compared them to their conventional observation-based counterpart (for details see Supplementary Methods 2). We expect model-based indicators to benefit from inter- and extrapolation of datapoints by being more robust to variability in sampling schedules. Correspondingly, we expect model-based indicators to be more suitable in case of combined data of original acute stress test studies.

### 2.2 Step 2: method validation with simulated data

To validate our novel model-based indicator method we leveraged a high temporal resolution simulated dataset that can provide “true” indicator values based on the full and original simulated cortisol response curves. This contrasts with indicator values that are based on low temporal resolution data according to specific sampling schedules that include sampling-related noise. The data were generated independent from our modeling approaches or the STRESS-EU dataset. The simulated dataset of n = 10,000 virtual individuals (50% females) was based on the stochastic differential equation (SDE) model by Miller et al. (2018). This model was implemented in R with package deSolve (Soetaert et al., 2010). The cortisol stress responses were simulated for the time period from −20 min to 80 min relative to TSST onset, with a sampling interval of 1 min. Simulations were performed by generating a random vector of initial (baseline) values and model parameters for each virtual individual. Those parameters were then submitted to a numeric differential equation solver that relied on the Euler-Maruyama method. The SDE model encompasses two error components: (1) white noise representing measurement error, and (2) red noise representing fluctuations of cortisol synthesis not accounted for by the simplified structural component of the gamma model (section 2.1.1). Thus, the SDE model preserves all features of actual stress test data. The simulated high-resolution cortisol stress response data with red noise but without white noise were used to calculate “true” indicator values that are free from sampling-related noise. “True” indicator values for AUCs were always calculated from 0 to 80 min relative to stressor onset, in correspondence with approximate return to baseline and the duration of saliva sampling in TSST protocols. Additionally, high-resolution cortisol stress response data with both red and white noise were downsampled to provide datasets according to any desired sampling schedule. These datasets mimic the infrequent sampling of real acute stress test studies. For example, we created twelve datasets according to twelve sampling schedules that together form a representative set of sampling schedules that may be applied in acute stress test studies (for details see Supplementary Methods 3). For every simulated participant within these datasets we then conventionally calculated observation-based indicators, but also applied the multilevel model and amplitude scaling model to derive model-based indicators. The use of simulated data allowed us to evaluate observation- and model-based indicator values against “true” indicator values to assess for each estimated indicator their accuracy (section 2.2.1), their stability with regards to different sampling schedules (section 2.2.2), their propriety in case of combined data (section 2.2.3), and their propriety in case of individual variability in sampling timings (section 2.2.4).

#### 2.2.1 Accuracy of indicators

First, general accuracy of indicator methods was evaluated per indicator by a comparison between the three methods. To evaluate each method, we assessed rank-order accuracy by calculation of Spearman correlations between “true” and estimated indicator values, because this relates to between-subject variation. Rank-order accuracy is arguably more important than individual error estimates, since research studies typically compare indicators of the cortisol response between groups of individuals or in association with other variables of interest. Spearman correlation coefficients were calculated for each of twelve sampling schedules from a representative set (for details see Supplementary Methods 3). We compared vectors of correlation coefficients (across all sampling schedules) between methods with three paired t-tests; we did not apply Fisher Z-transformations here given effect size inflation with high correlation coefficients. Correlation coefficients were however still Fisher Z-transformed to conduct Z-tests and determine per indicator for each sampling schedule the most accurate method.

#### 2.2.2 Stability of indicators

If an indicator method produces a similar indicator value regardless of the applied sampling schedule, it demonstrates the desired cross-sampling-schedule stability for research on combined datasets. Each conventional observation-based indicator is expected to have weaknesses that may threaten indicator stability; these could potentially be addressed by using model-based indicators. To determine whether this is true, we systematically investigated expected weaknesses using sets of sampling schedules that differ in one specific factor: sampling duration, sampling frequency, or peak timepoint (for details see Table S1 in Supplementary Methods 4). Here, higher indicator stability is indicated by larger rank-order correspondence across sampling schedules, which is calculated as higher Spearman correlations between estimated indicator values of different sampling schedules. To quantify differences between methods with regard to indicator stability, vectors of correlation coefficients from each set of sampling schedules were compared between methods with paired t-tests.

#### 2.2.3 Suitability of indicators in case of combined data

Subsequently, we simulated combined data. Firstly, per set of sampling schedules that differs in only one specific factor (described above), each simulated participant was randomly allocated with a sampling schedule from the respective set of schedules. We then calculated observation-based and model-based indicators for all simulated participants. With variability in sampling duration, observation-based indicators were calculated in two ways: (1) neglecting differences in sampling duration, and (2) the latest timepoint that is common across all participants was used as cut-off. This process of combined data simulation was repeated ten times for each set of sampling schedules to achieve robust results. Secondly, since real-world examples of combined data often show variation in more than one factor (e.g. sampling duration *and* sampling frequency), each indicator method was also evaluated with more typical data examples. Two combined datasets with variation in sampling frequency, duration and peak sample timing were simulated as described above, according to (1) twelve representative sampling schedules, and (2) five high-variability sampling schedules (for details see Supplementary Methods 3). To investigate applicability of each indicator method to combined data, per indicator we reported each method's rank-order accuracy (Spearman correlation between “true” and estimated indicator values); these values also underwent three permutation tests for pairwise comparisons between methods. Then we performed analyses to provide insight into the impact of indicator (in)stability to a certain factor (e.g. sampling duration) on rank-order accuracy when aggregating data. Higher decrease in rank-order accuracy with combined data is signaled by a larger difference between the combined data rank-order accuracy and the average rank-order accuracy from single sampling schedules within the set. For comparison, we therefore also calculated Fisher Z-transformed, averaged correlation coefficients of rank-order accuracy for each set of sampling schedules. Finally, we assessed Spearman correlations of indicator values with sampling duration.

#### 2.2.4 Suitability of indicators in case of individual variability in sampling timings

We additionally simulated two datasets as if they belong to acute stress test studies that faced delays in their study procedures and as such each show individual variation in sampling timings (for details see Supplementary Methods 5). This resulted in data with medium individual variability in sampling schedules according to sampling timepoints M (*SD*): [0 (0.0), 21.7 (1.5), 43.9 (2.2), 66.1 (2.6), 80], and data with high individual variability in sampling schedules according to sampling timepoints M (*SD*): [0 (0.0), 31.8 (5.0), 60.4 (5.6)]. Conventional observation-based indicators were calculated with average sampling timepoints across participants, however for the model-based indicators, data inter- and extrapolation allowed the use of individual sampling timepoints (with a precision of 1 min). Indicator methods were then evaluated in terms of rank-order accuracy as described previously.

### 2.3 Step 3: method application to independent acute stress test studies

Two observational datasets were used to apply our novel model-based indicator methods to real-world acute stress test data. The first dataset comprises a local dataset of students who completed a modified version of the SECPT procedure and a matched control procedure according to a within-subjects design (Tutunji et al., 2024). Data was collected as part of an fMRI study and exhibits individual variation with regard to exact timings of cortisol samples (relative to stressor onset). Baseline values were corrected as previously described (section 2.1.1), and procedures without corrected baseline value were removed from the dataset. Afterwards, M (*SD*) timepoints of saliva sampling were 0 (0.0), 8.1 (3.9), 37.0 (5.3), 81.2 (9.4), and 153.7 (11.0) minutes relative to stressor onset. The last timepoint was discarded for the current study. After the exclusion of two ±5 SD outlier cortisol values, and exclusion of five procedures without sampling timepoints from 15 to 45 min after stressor onset, the final dataset contained 540 salivary cortisol samples from n = 73 participants (45 F, age *M*(*SD*) = 19.5(1.6)) who completed 68 stress and 67 control procedures in total. The second dataset consists of n = 80 young adult participants [43 F, age *M* (*SD*) = 23.6 (4.1)] (Voulgaropoulou et al., 2022). Participants completed a MAST stress or control condition procedure according to a between-subjects study design (n = 40 for each condition). Salivary cortisol was sampled at −10, 0, 10, 20, 30, 40, and 50 min relative to stressor onset; the first sample (prior to baseline) however was not used in the current study. This dataset did not contain any missing data or ±5 SD outlier cortisol values. Of note, this dataset was added to the STRESS-EU database in December 2023 after the data for the current study was obtained, it is therefore independent of the STRESS-EU data used for model development.

Both models were applied to the two acute stress test datasets to illustrate the opportunities of our novel model-based indicator approach. Although we argue that conventionally calculated indicators show their limitations, they are the current gold standard in the field of stress research; associations between conventional observation-based and novel model-based indicators would therefore be desired and could provide some form of external validation. Therefore, Spearman correlations between conventional and model-based indicators are considered per condition (stress versus control) and per dataset, as well as for the combined dataset.

## 3 Results

### 3.1 Step 1: method development with STRESS-EU data – model fits and performances

**Fig. 2:**
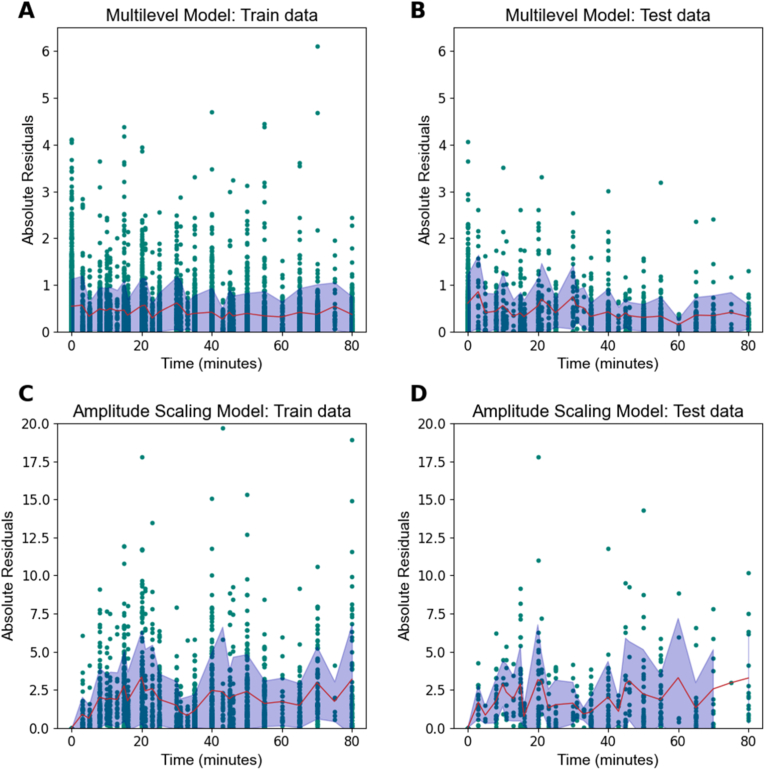
Absolute errors in STRESS-EU train and test datasets from each train model. Points show each observation in the dataset, whereas the solid red line and shading reflect the mean and standard deviation. **Note**: Different scaling of y-axes indicates that the multilevel model produces significantly lower error values.

**Fig. 3:**
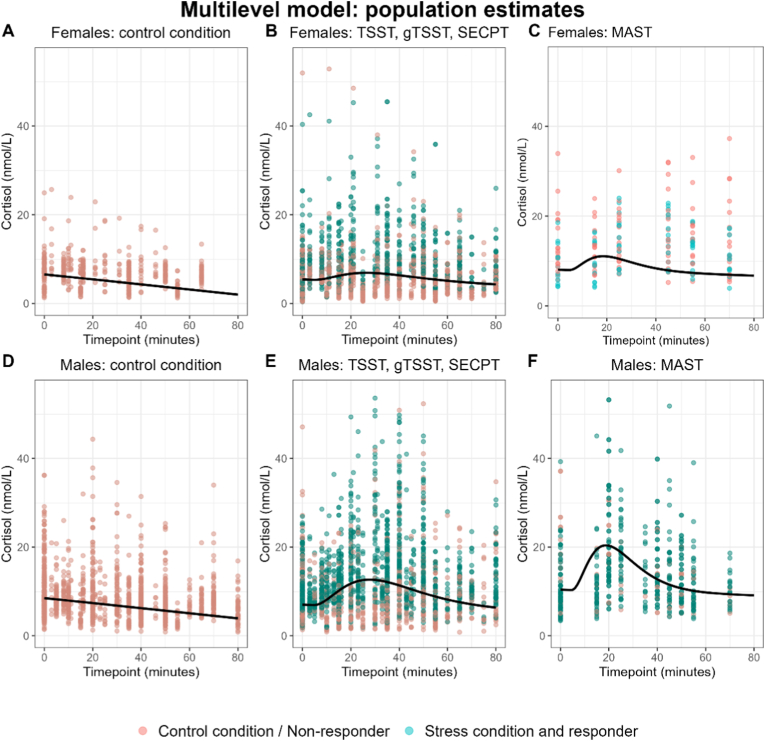
Cortisol stress response ‘population estimates’ from the multilevel model (line) with observed values (points) for (A) Females who participated in a no-stress control condition, (B) Females who participated in TSST, gTSST, or SECPT, (C) Females who participated in MAST, (D) Males who participated in a no-stress control condition, (E) Males who participated in TSST, gTSST or SECPT, and (F) Males who participated in MAST.

**Fig. 4:**
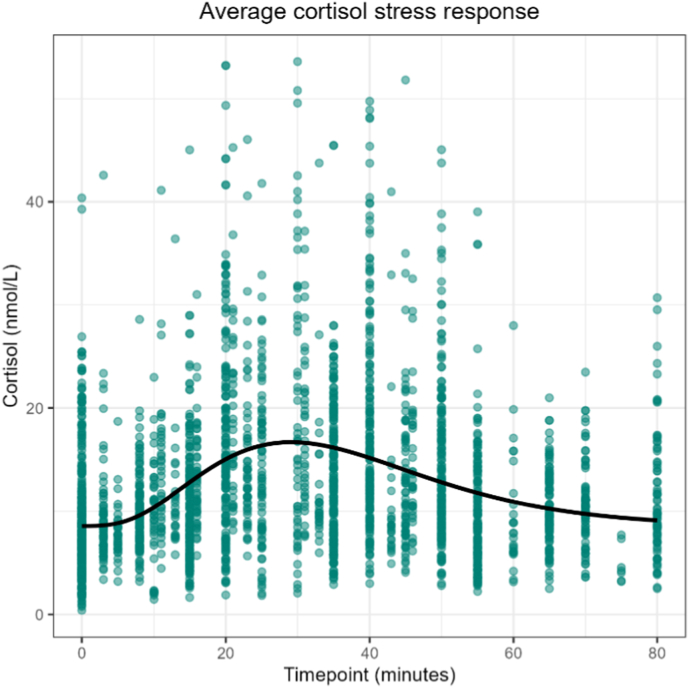
The population average cortisol response curve (solid line) which was fitted on observations (datapoints) from stress condition responder participants. In the amplitude scaling model, the amplitude of this curve was scaled for each individual to model individual cortisol stress response curves.

After fitting each model on STRESS-EU data, their accuracy is signaled by mean absolute error (MAE) values (i.e. mean of absolute differences between observed and predicted cortisol values per participant) and bias values (i.e. mean of observed minus predicted cortisol values per participant) averaged across participants. For both models, MAE values from the training and test datasets are displayed in Fig. 2 (for bias values see Fig. S3 in Supplementary Results 1)**.** With the multilevel model, MAE values were 0.43 nmol/L for the training data and 0.44 nmol/L for the test data. Application of the amplitude scaling model resulted in MAE values of 1.74 nmol/L for the training data and 1.66 nmol/L for the test data. Since these MAE values do not indicate overfitting to the training dataset, we applied the selected modeling approaches to the full (training + test) dataset to create the final models. Fig. 3 shows the final ‘population estimates’ of the multilevel model after application of model fitting to the full dataset. Since the multilevel model considered gender and stress paradigm as covariate factors, separate cortisol response curves were generated based on these factors. Fig. 4 and shows the average cortisol stress response curve as derived from the full data subset of stress condition responder participants that was used for the amplitude scaling model (see also Fig. S4 in Supplementary Results 2).

### 3.2 Step 2: method validation with simulated data – comparing cortisol summary indicators

To address to what extent conventional observation-based indicators fall short, and model-based indicators improve suitability to combined datasets, we leveraged a high temporal resolution simulated dataset that could provide the “true” indicator values. These “true” indicator values were based on the full and original simulated cortisol response curves. For information, correlations between such “true” indicator values are presented in Fig. S5 (see Supplementary Results 3). These “true” indicator values allowed evaluation of observation-based and model-based indicator estimates derived from downsampled data that reflect realistic, sparse-sampling acute stress test datasets (e.g., sampling every 10 min).

#### 3.2.1 Descriptive statistics

The mean “true” indicator values from the simulated dataset were 189.1 (*SD* = 225.5) nmol/L∗min for AUCi, 538.0 (*SD* = 258.6) nmol/L∗min for AUCg, 4.96 (*SD* = 5.43) nmol/L for reactivity and 7.21 (*SD* = 5.62) nmol/L for maximum increase. Descriptive statistics of estimated indicator values according to a representative set of twelve sampling schedules (for details see Supplementary Methods 3) are reported in Table 1, and per sampling schedule in Tables S2–4 (Supplementary Results 4). These descriptives include means and standard deviations across sampling schedules of (1) mean indicator estimates across participants (M_M_, SD_M_), (2) MAE values across participants (M_MAE_, SD_MAE_): mean absolute differences between “true” and estimated indicator values, and (3) mean bias across participants (M_bias_, SD_bias_): mean of “true” indicator minus estimated indicator values.

**Table 1:** Descriptive statistics and rank-order accuracy of cortisol stress response indicators as derived from the simulated dataset according to the three methods, averaged across a representative set of twelve sampling schedules.

Indicator	Model performance measure	OBS	MM	ASM
AUCi	M_M_ (*SD*_M_)	168.2 (36.7)	169.9 (15.1)	197.3 (8.3)
	M_MAE_ (*SD*_MAE_)	64.0 (24.8)	62.8 (8.3)	55.6 (5.4)
	M_bias_ (*SD*_bias_)	20.9 (36.7)	19.1 (15.1)	−8.5 (8.3)
	M*r*_*s*,single_	0.905	*0.883*∗	0.902
AUCg	M_M_ (*SD*_M_)	426.1 (106.5)	553.9 (14.1)	546.3 (8.3)
	M_MAE_ (*SD*_MAE_)	120.6 (96.9)	44.6 (20.9)	49.9 (8.3)
	M_bias_ (*SD*_bias_)	111.9 (106.5)	−15.9 (14.1)	−8.3 (8.3)
	M*r*_*s*,single_	**0.964**∗	0.952	0.950
Reactivity	M_M_ (*SD*_M_)	4.70 (0.35)	4.47 (0.10)	5.16 (0.22)
	M_MAE_ (*SD*_MAE_)	1.05 (0.22)	1.05 (0.17)	1.20 (0.17)
	M_bias_ (*SD*_bias_)	0.26 (0.35)	0.49 (0.10)	−0.20 (0.22)
	M*r*_*s*,single_	**0.932**∗	0.932	0.920
Maximum increase	M_M_ (*SD*_M_)	6.63 (0.74)	6.14 (0.33)	5.44 (0.19)
	M_MAE_ (*SD*_MAE_)	1.25 (0.4)	1.43 (0.29)	2.07 (0.18)
	M_bias_ (*SD*_bias_)	0.57 (0.74)	1.07 (0.33)	1.77 (0.19)
	M*r*_*s*,single_	0.890	0.896	*0.817*∗

**Abbreviations:** ASM = amplitude scaling model, AUCg = Area Under the Curve with respect to Ground, AUCi = Area Under the Curve with respect to Increase, MM = multilevel model, OBS = conventional observation-based method.

**Descriptive and test statistics:** M_M_ (*SD*_M_) = mean (standard deviation) of mean indicator estimates, M_MAE_ (*SD*_MAE_) = mean (standard deviation) of mean absolute error values, M_bias_ (*SD*_bias_) = mean (standard deviation) of mean bias (“true” − estimated values), M*r*_*s,*single_ = mean Spearman correlation coefficient of “true” with estimated values from application of single schedules to data.

**Superscripts:** Asterisks denote statistically significant (*p* < .05) results from paired t-tests that compared vectors of Spearman correlation coefficients between “true” and estimated indicator values. These indicate a difference in rank-order accuracy between methods. ,∗*p* < .05.

**Note:** The values indicate mean Spearman correlation coefficients between “true” and estimated indicator values (M*r*_*s*,single_). Based on results from statistical testing, **bold** faced numbers present the most favourable method, whereas *italics* numbers present the least favourable method.

#### 3.2.2 Accuracy of indicators

We first compared observation-based indicators (i.e., conventional method) and model-based indicators (i.e., from the multilevel model and amplitude scaling model) in terms of their general accuracy across a representative set of twelve sampling schedules (for details see Supplementary Methods 3). Accuracy of each indicator was assessed with respect to rank order, that is using Spearman *r* correlations between “true” and estimated indicator values. Subsequently, general indicator accuracy was compared between methods. We then also examined differences in rank-order accuracy per sampling schedule. Correlation coefficients are reported in Table 1 (M*r*_*s*,single_) and Table S5 (i.e., results per sampling schedule; see Supplementary Results 5). Notably, very sparse sampling schedules (e.g. [0, 30]) descriptively show lower accuracy for all methods. We also found some differences in general rank-order accuracy between methods, and conventional observation-based indicators scored generally slightly better than model-based indicators. However, these differences do not appear to have a relevant effect size: overall all indicators showed high rank-order accuracy across all three methods. This raised the question if observation-based indicators from the conventional method show expected weaknesses related to specific factors of variation in sampling schedules (sampling duration, sampling frequency and peak timepoint), and if so, whether these could be addressed by using model-based indicators.

#### 3.2.3 Stability of indicators

Results of cross-sampling-schedule stability, investigated systematically across schedules that differ in one specific factor (sampling duration, sampling frequency, or peak timepoint), are presented in Table 2 (M*r*_*s*,stability_). The three methods generally show similarly high levels of indicator stability, especially with regard to AUC indicators (generally M*r*_*s*,stability_ > 0.9). Any differences, which are usually in favor of the amplitude scaling model, are small and appear minimally relevant in terms of their effect size.

**Table 2:** Results of systematic investigation of cortisol stress response indicator stability with respect to sampling duration, sampling frequency and peak timepoint, from simulated data, according to the three methods.

Factor	Indicator	Set	Method		
			OBS	MM	ASM
Duration	AUCi	(1)	0.973∗	*0.896*∗	**0.995**∗
	AUCg	(1)	0.980∗	*0.942*∗	**0.997**∗
	Maximum increase	(1)	0.964	0.896	**0.991**∗
Frequency	AUCi	(1)	0.943	*0.905*∗	0.938
		(2)	0.967	0.949	0.978
	AUCg	(1)	0.952	0.944	0.959
		(2)	0.974	0.965	0.987
	Maximum increase	(1)	0.792	0.814	0.894
		(2)	0.911	0.921	**0.968**∗
Peak timepoint	Reactivity	(1)	0.908	*0.892*∗	0.908
		(2)	0.908	*0.893*∗	**0.922**∗
		(3)	0.905∗	0.886∗	0.906
	Maximum increase	(1)	0.822	*0.786*∗	**0.863**∗
		(2)	0.839	0.839	**0.872**∗
		(3)	0.843	0.826	0.847

**Abbreviations:** AUCg = Area Under the Curve with respect to Ground, AUCi = Area Under the Curve with respect to Increase, ASM = amplitude scaling model, NA = not applicable, MM = multilevel model, OBS = conventional observation-based method.

**Superscripts:** Asterisks denote statistically significant (*p* < .05) results from paired t-tests that compared vectors of Spearman correlation coefficients between estimated indicator values of different sampling schedules. These indicate a difference in indicator stability between methods.

**Note:** The values indicate mean Spearman correlation coefficients between estimated indicator values of different sampling schedules (M*r*_*s*,stability_). Based on results from statistical testing, **bold** faced numbers present the most favourable method, whereas *italics* numbers present the least favourable method.

#### 3.2.4 Suitability of indicators in case of combined data

In the next step, we simulated combined data and used the same sets of sampling schedules (that differ in one factor only) in a systematic evaluation of indicator (in)stability. This is important, because combined data would require an indicator method that shows stability in case of variability in sampling schedules, without correlation with sampling duration, to prevent bias in analyses. Table 3 reports on the decrease in rank-order accuracy with combined data (M*r*_*s*,comb_ versus M*r*_*s*,single_). For the three different factors of variation, we applied permutation testing to directly compare the amplitude scaling model-based (ASM-based), multilevel model-based (MM-based), and conventional observation-based (OBS-based) indicators in terms of rank-order accuracy.

**Table 3:** Results of systematic investigation of rank-order accuracy of cortisol stress response indicators with respect to sampling duration, sampling frequency and peak timepoint, from simulated combined data, according to the three methods.

Factor	Indicator	Set	Method			
			OBS	OBS-s	MM	ASM
Duration	AUCi	(1)	0.900∗ (0.907)	*NA* (0.871)	*0.877*∗ (0.880)	**0.915∗** (0.915)
	AUCg	(1)	*0.873*∗ (0.973)	*NA* (0.939)	0.955∗ (0.956)	**0.961∗** (0.961)
	Maximum increase	(1)	**0.910∗** (0.913)	*NA* (0.896)	0.889∗ (0.890)	*0.822*∗ (0.822)
Frequency	AUCi	(1)	0.890 (0.897)		*0.871*∗ (0.875)	0.891 (0.896)
		(2)	**0.912∗** (0.913)		*0.890*∗ (0.891)	0.910∗ (0.911)
	AUCg	(1)	0.960 (0.966)		0.959 (0.976)	*0.947*∗ (0.951)
		(2)	**0.971∗** (0.971)		0.963∗ (0.962)	*0.957*∗ (0.957)
	Maximum increase	(1)	0.751∗ (0.811)		**0.825∗** (0.840)	*0.737*∗ (0.740)
		(2)	0.881∗ (0.897)		**0.893∗** (0.896)	*0.795*∗ (0.796)
Peak timepoint	Reactivity	(1)	0.896∗ (0.904)		*0.880*∗ (0.886)	**0.899∗** (0.904)
		(2)	**0.896**∗ (0.904)		0.893 (0.896)	*0.833*∗ (0.889)
		(3)	0.880 (0.889)		0.882 (0.886)	0.880 (0.886)
	Maximum increase	(1)	0.771∗ (0.792)		*0.746*∗ (0.759)	**0.775∗** (0.776)
		(2)	0.804∗ (0.823)		**0.823∗** (0.827)	*0.742*∗ (0.744)
		(3)	*0.727*∗ (0.748)		**0.759∗** (0.767)	0.735∗ (0.736)

**Abbreviations:** AUCg = Area Under the Curve with respect to Ground, AUCi = Area Under the Curve with respect to Increase, ASM = amplitude scaling model, NA = not applicable, MM = multilevel model, OBS = conventional observation-based method, OBS-s = conventional observation-based method only including observations according to shortest sampling schedule.

**Superscripts:** Asterisks denote statistically significant results (*p* < .05) from pairwise permutation tests that compared vectors of Spearman correlation coefficients between “true” and estimated indicator values across ten combined datasets (*p* < .05). These indicate a difference in rank-order accuracy for combined datasets between methods.

**Note:** The values indicate mean Spearman correlation coefficients between “true” and estimated indicator values across ten combined datasets (M*r*_*s*,comb_). Based on statistical testing, **bold** faced numbers present the most favourable method, and *italics* the least favourable method. The values between brackets indicate mean Spearman correlation coefficients between “true” and estimated indicator values across applications of single sampling schedules to data (for comparison, to signal the loss of accuracy with combined data).

**Note (2):** This table also includes results for the application of the conventional observation-based method while only including observations according to the shortest sampling schedule (OBS-s); this fully harmonizes sampling schedules in terms of sampling duration. Without variation in sampling schedules, there is no impact of data combination (i.e., *NA*).

Firstly, we tested combined data with variations in sampling duration, and assessed the three methods for AUCi, AUCg and maximum increase. Regarding AUCi, only the OBS-based indicator showed a small correlation with sampling duration (M*r*_s,sd_ = 0.089, *p* < .001), but this appeared to have little impact on its accuracy (M*r*_*s*,single_ = 0.907 versus M*r*_*s*,comb_ = 0.900). Overall, ASM-based AUCi showed highest accuracy, while the MM-based AUCi showed lowest accuracy. Regarding AUCg, the OBS-based indicator moderately correlated with sampling duration (M*r*_*s,*sd_ = 0.414, *p* < .001). Consequently, the accuracy of OBS-based AUCg decreased with combined data (M*r*_*s*,single_ = 0.973 versus M*r*_*s*,comb_ = 0.873), so that the OBS method was least accurate. It was outperformed by both MM-based AUCg and ASM-based AUCg, which did not correlate with sampling duration. Regarding maximum increase, the OBS-based indicator again showed a small correlation with sampling duration (M*r*_*s,*sd_ = 0.075, *p* < .001), while the MM-based indicator showed a small negative correlation (M*r*_*s,*sd_ = 0.060, *p* < .001). Nevertheless, the OBS-based indicator was still more accurate than both model-based indicators.

Secondly, for the same three indicators, we tested combined data with variations in sampling frequency. Regarding AUCi and AUCg, the OBS-based indicators showed similar accuracy or slightly outperformed the model-based indicators. These differences were however small, and therefore, likely irrelevant. Regarding maximum increase, in the first set of sampling schedules, accuracies of the OBS-based and ASM-based indicators were relatively low (M*r*_*s*, comb_ < 0.8); in both sets, the MM-based indicator performed best.

Thirdly, we tested combined data with variations in peak timepoint to evaluate the three methods for the maximum increase and reactivity indicators. The results depended on the sampling duration of the set of schedules. Regarding maximum increase, accuracy was relatively low (M*r*_*s*, comb_ < 0.8) for two of the three sets of sampling schedules. The MM-based indicator was preferred, except when sampling was limited to the reactivity phase (set 1), that is, discontinued after the peak timepoint; then it was slightly outperformed by the ASM-based indicator. Reactivity showed a similar pattern of results, but showed generally higher accuracy and equally favourable performance of the OBS-based indicator.

In summary so far, when combined data contained schedules that varied in sampling duration, the conventional observation-based method specifically showed weakness to AUCg: this indicator was correlated to sampling duration. Either model-based AUCg indicator provided an appropriate solution. Overall, the ASM-based indicator for AUCi was slightly more stable and accurate than the other AUCi indicators. Furthermore, the reactivity indicator showed similar accuracies for all three methods, which were all acceptable; the maximum increase indicator, however, showed relatively lower accuracy for all three methods.

Subsequently, we evaluated indicators by using more typical examples of combined data. After all, the factors that were addressed in the systematic investigation that is reported above may interact if combined data differs on more than one factor, for instance with variability in sampling duration *and* sampling frequency. This is usually true for larger combined datasets of individual studies – for example, in the STRESS-EU database. Table 4 shows results from simulating combined datasets according to representative sample (RS) and high variability (HV) sets of sampling schedules (for details see Supplementary Methods 3).

**Table 4:** Rank-order accuracy of cortisol stress response indicators, from simulated combined data, according to the three methods.

Indicator	Data	Method		
		OBS	MM	ASM
AUCi	RS	0.876 (0.905)	0.877 (0.893)	**0.901**∗ (0.902)
	HV	*0.847*∗ (0.897)	0.861∗ (0.884)	**0.896**∗ (0.897)
AUCg	RS	*0.806*∗ (0.964)	0.949∗ (0.960)	**0.950**∗ (0.950)
	HV	*0.723*∗ (0.958)	0.932 (0.960)	**0.946**∗ (0.949)
Reactivity	RS	**0.929**∗ (0.932)	0.926∗ (0.932)	*0.919*∗ (0.920)
	HV	0.926 (0.929)	0.926 (0.932)	*0.919*∗ (0.920)
Maximum increase	RS	0.870∗ (0.890)	**0.884**∗ (0.896)	*0.816*∗ (0.817)
	HV	0.855∗ (0.879)	**0.864**∗ (0.882)	*0.817*∗ (0.817)

**Abbreviations:** ASM = amplitude scaling model, AUCg = Area Under the Curve with respect to Ground, AUCi = Area Under the Curve with respect to Increase, HV = high variability data, MM = multilevel model, OBS = conventional observation-based method, RS = representative sample data.

**Superscripts:** Asterisks denote statistically significant results (*p < *.05) from pairwise permutation tests that compared vectors of Spearman correlation coefficients between “true” and estimated indicator values across ten combined datasets (*p* < .05). These indicate a difference in rank-order accuracy for combined datasets between methods.

**Note:** The values indicate mean Spearman correlation coefficients between “true” and estimated indicator values across ten combined datasets (M*r*_*s*,comb_). Based on statistical testing, **bold** faced numbers present the most favourable method, and *italics* the least favourable method. The values between brackets indicate mean Spearman correlation coefficients between “true” and estimated indicator values across applications of single sampling schedules to data (for comparison, to signal the loss of accuracy with combined data).

The results confirmed that OBS-based AUCg shows moderate-to-strong correlations with sampling duration (RS M*r*_*s*,sd_(9998) = 0.489, *p* < .001; HV M*r*_*s*,sd_(9998) = 0.615, *p* < .001). As a consequence, it showed substantial decreases in accuracy with combined data (RS M*r*_*s*,single_(9998) *=* 0.964 versus M*r*_*s*,comb_(9998) = 0.806, HV M*r*_*s*,single_(9998) = 0.958 versus M*r*_*s*,comb_(9998) = 0.723). OBS-based AUCi also showed weak correlations with sampling duration (RS M*r*_*s*,sd_(9998) = 0.157, *p* < .001; HV M*r*_*s*,sd_(9998) = 0.239, *p* < .001). Overall, permutation tests indicated that both OBS-based indicators AUCg and AUCi are less accurate than either of the model-based AUC indicators (all *p* < .001), although this difference was much smaller for AUCi. Of note, MM-based AUC indicators usually showed a very weak correlation in opposite direction across participants (RS AUCi M*r*_*s*,sd_(9998) = −0.052, *p* < .001; HV AUCi M*r*_*s*,sd_(9998) = −0.090, *p* < .001; HV AUCg M*r*_*s*,sd_(9998) = −0.073, *p* < .001). ASM-based AUC indicators showed no correlation with sampling duration and (minimally) higher accuracy for both AUCi and AUCg, and thus appeared most suitable to apply to combined data. With regard to reactivity and maximum increase indicators, MM-based indicators showed higher accuracy than other indicators. Although ASM-based reactivity and maximum increase showed lower decreases in accuracy with combined data, its estimates were least accurate of the three methods. Especially for reactivity, differences between methods were however small and may therefore be irrelevant.

#### 3.2.5 Suitability of indicators in case of individual variation in sampling timings

Variation in sampling schedules is not contained to combined data, but may also exist within data from single studies that were logistically challenging. Whether model-based indicators then show advantages in comparison to conventional observation-based indicators was assessed with simulations that induced individual variability in sampling (see Supplementary Methods 5). Results from these analyses are reported in Supplementary Results 6. Generally, rank-order accuracy was high with minimal differences between methods; conventional observation-based indicators appeared thus reasonably applicable to data with individual differences in sampling timings.

### 3.3 Method application to independent acute stress test studies

In the previous sections we leveraged simulated data to compare model-based with conventional indicators. Although these generated data are supposed to reflect all characteristics of acute stress test data, real data may be more capricious due to between-study differences in protocols, anticipatory stress responses or additional real-world measurement noise. Furthermore, the current data simulations were specifically based on data from TSST stress procedures. We therefore applied indicator methods to two independent acute stress test datasets from different types of test, that also included no-stress control procedures (see section 2.3). Table 5 presents correlations for model-based indicators with their conventional observation-based counterpart.

**Table 5:** Spearman correlation coefficients between model-based indicators and conventional indicators within and across two independent acute stress test datasets.

Indicator	Method	(1) Tutunji et al.			(2) Voulgaropoulou et al.			All data
		Stress	Control	All	Stress	Control	All	
AUCi	MM	0.873	0.832	0.853	0.943	0.883	0.910	0.866
	ASM	0.970	0.956	0.973	0.999	0.995	0.998	0.975
AUCg	MM	0.965	0.979	0.972	0.968	0.978	0.967	0.916
	ASM	0.922	0.959	0.946	0.980	0.986	0.985	0.890
Reactivity	MM	0.926	0.736	0.870	0.925	0.964	0.945	0.907
	ASM	0.994	0.994	0.996	0.232^ns^	−0.098^ns^	0.086^ns^	0.634
Maximum increase	MM	0.847	0.617	0.755	0.130^ns^	0.257^ns^	0.249	0.506
	ASM	0.854	0.736	0.807	0.940	0.873	0.922	0.849

**Abbreviations:** AUCg = Area Under the Curve with respect to Ground, AUCi = Area Under the Curve with respect to Increase, ASM = amplitude scaling model, MM = multilevel model.

**Superscripts:**^ns^ Spearman correlation is not statistically significant (*p* > .05).

The results showed that associations of model-based AUC indicators with observation-based AUC indicators were consistently high for single (stress/control) groups as well as combined data. This is regarded as some form of external validation, since observation-based indicators are currently the gold standard in our field. Some inconsistencies should, however, be noted and explained. Addressing the correlations between all indicators supports the interpretation of these findings (see Figs. S6–10 in Supplementary Results 7). Firstly, the second independent dataset (Voulgaropoulou et al.) showed no correlation of ASM-based reactivity with OBS-based reactivity. In both independent datasets, ASM-based reactivity perfectly correlates and thus fully reflects ASM-based AUCi. While in the simulated dataset as well as the first independent dataset (Tutunji et al.), reactivity and AUCi indicators are highly correlated (*r* > 0.9), the second independent dataset (Voulgaropoulou et al.) does not show these correlations. This lack of correlation between (OBS-based) reactivity and (OBS-based) AUCi in this dataset underlies the lack of correlation between OBS-based reactivity and ASM-based reactivity (i.e., ASM-based AUCi). Secondly, the second independent dataset (Voulgaropoulou et al.) showed no correlation of MM-based maximum increase with OBS-based maximum increase. In these data (Voulgaropoulou et al.)**,** MM-based maximum increase reflected conventional peak timepoint instead.

## 4 Discussion

In this study, we developed a novel method regarding cortisol summary indicators, which can be applied to combined data. This method was developed in three steps. In the first step, we developed two statistical modeling approaches using combined data from acute stress test studies in the STRESS-EU database. The first approach, the multilevel model, estimated from the full STRESS-EU dataset, is characterized by parsimonious estimation of individual parameters and additionally considered population estimates related to gender and type of acute stress test. The second approach, the amplitude scaling model, applied amplitude scaling of a population average cortisol response that was estimated based on cortisol responder participants from stress conditions in the STRESS-EU database, thus reflecting a simplified model of the cortisol stress response. Both models were applied to individual cortisol stress response curves to derive model-based summary indicators of the cortisol stress response – AUCi, AUCg, reactivity, maximum increase. In the second step, we investigated with simulated data if these model-based indicators are more robust to between-study sampling variation than conventional observation-based indicators. In the third step, these methods were applied to independent acute stress test datasets to provide external validation. Using simulated data (step 2), we were able to demonstrate that the conventional (i.e., non-model-based) method mainly falls short in estimation of AUC indicators when combined data contains variation in sampling duration. As expected, conventional observation-based AUC indicators were positively correlated with sampling duration and this negatively impacted accuracy for combined data. This was most prominent for the AUCg metric. Statistical modeling extrapolates data, and as such offers equal datapoints across all participants to-be-used for the calculation of summary indicators, thereby eliminating positive correlations with sampling duration. While both model-based indicators provide an appropriate solution for the application of AUC indicators to combined data with variations in sampling schedules, the use of the amplitude scaling model is recommended: this method was slightly more accurate, and can be easily and readily be applied by other researchers by using the formula of the population average cortisol curve (Supplementary Methods 1) and the code from the supplemented Python notebook scripts.

Our findings also indicated that from conventional observation-based indicators, specifically the reactivity indicator actually appears suitable for most types of combined data. Importantly, however, without an observation at the determined peak timepoint, conventional reactivity requires derivation from the timepoint(s) closest in time, which may lead to slightly decreased accuracy. Therefore, reactivity indicators are most suitable for combined datasets with low variability in the peak timepoint. In the case of very sparse sampling schedules without early recovery observations (e.g. only baseline and 30 min post-stressor onset), all methods struggle with accurate estimation of the cortisol stress response; this impacted estimation of the maximum increase indicator the most. The results showed that a multilevel model-based maximum increase metric is a suitable solution only if late recovery observations are available (e.g. 80 min post stressor onset). In summary, we find that application of conventional reactivity and maximum increase indicators may be suitable for some forms of combined data, but with some lack of consistency. The added value of this model-based approach is that it enables researchers to select the cortisol response indicator(s) that are most appropriate for their specific research question and data characteristics, rather than being restricted to a single observation-based metric. By applying conventional and model-based methods to two independent datasets, we provide an initial external validation for use of model-based indicators, including in no-stress control procedures. Moreover, correlational analyses clarified the characteristics of model-based indicators when applied to real-world data. Amplitude scaling model-based reactivity and AUCi measures scale linearly with one another, as both are determined only by fitting the amplitude of the response. The AUCi measure based on the amplitude-scaling model therefore does not reflect additional information regarding the shape of the response, but is included here because it is one of the most commonly used cortisol response measures. Note, however, that amplitude estimation is based on all available timepoints rather than solely on the peak. Amplitude scaling model-based reactivity and AUCi therefore more closely correspond to conventional observation-based AUCi, which also combines measures across all timepoints. Besides that, it should be noted that the interpretation of the maximum increase indicator depends on the experimental context. When there is a stress response, maximum increase indicators closely align with reactivity. However, since the definition of maximum increase constrains this measure to positive values only, maximum increase indicators inversely scale with the negative amplitude when a control condition or non-response is characterized by a decline in cortisol. All in all, we recommend the use of model-based AUC indicators, because it behaves most robustly under different circumstances. Note that this attempt at external validation should be interpreted in the light of limitations of ‘gold standard’ conventional indicators. In the future, further validation may be achieved from leveraging densely sampled empirical studies, as this would enable fitting models to down-sampled real-world data.

As expected, the multilevel model showed a much more accurate fit to the cortisol stress response than the simplified amplitude scaling model, although accurate fit was not a guarantee to obtain accurate indicator estimates. That is, despite its poorer fit to the cortisol response curve, the amplitude scaling model-based AUCi showed slightly higher accuracy in terms of rank order across individuals than multilevel model-based AUCi. Although amplitude scaling model-based AUCi and conventional AUCi both utilize cortisol levels at t = 0 min as baseline value, in our simulations this value of t = 0 min was still different from the “true” baseline value due to white noise (reflecting measurement noise). The multilevel model treats the t = 0 min value as a noisy measurement, but nevertheless did not outperform amplitude scaling model-based AUCi. In some cases, for example with high anticipatory stress, we speculate that this characteristic of the multilevel model may provide an additional advantage.

Beyond the purpose of the current study, the multilevel model may also provide opportunity to characterize individual differences in the temporal dynamics of the cortisol stress response. Previous studies have already applied multilevel growth curve models to extract latent growth factors for cortisol baseline, response and recovery (Lopez-Duran et al., 2014; Schlotz et al., 2011). In a new approach, psi values from random effects within a multilevel model could shed light on individual differences in the shape and temporal dynamics of the cortisol response. However, the application of this method to combined data should be carefully considered, as variation in sampling schedules and acute stress test procedures may impact random effect parameter estimates and confound the effect of interest. Nonetheless, for data from single acute stress test studies, the multilevel model might provide a new avenue to further explore the dynamics of the cortisol stress response.

The current study has several limitations. First of all, the majority of participants completed the (g)TSST. Our findings may not generalize well to other types of acute stress tests, particularly those without a social-evaluative component. Second, study protocols were viewed as uniform within each category of acute stress tests. Due to the limitations of the data within the database, we were unable to address protocol variations within a category, for example with regard to the duration of the stressor. Although we could not assess to what extent such variations increase heterogeneity in stress responses, it should be noted that this heterogeneity is nonetheless modeled by the multilevel model. Third, the multilevel model estimates individual cortisol response curves conditional on gender and laboratory acute stress test, which are included as covariates to improve curve estimation and prediction. As a consequence, cortisol indicators derived from these curves (e.g., AUC) should not be used in subsequent analyses to re-test effects of gender or stress-test type, as these variables already influence the individual estimates through shrinkage. Downstream inference on other predictors (e.g., age) remains valid only if they are uncorrelated to these covariates, and corresponds to testing their association with cortisol indicators adjusted for gender and stress-test differences. Researchers should be aware that their analyses may be invalided if their predictors are unknowingly related to these covariates, and may therefore prefer the use of the amplitude scaling model. Fourth, although the gamma distribution fits well to the population average, it may not always fit a given individual's cortisol stress response exactly. From a theoretical perspective, it may be particularly unsuitable to model individual variation in the recovery phase. Although it is suggested that cortisol reactivity and recovery constitute two independent components (Degering et al., 2023; Ji et al., 2016; Linden, W., Earle, T. L., Gerin, W., & Christenfeld, 1997), the shape parameter α from the gamma distribution does not only affect kurtosis, but also the skewness of the distribution. This means that it cannot regulate the slope of cortisol recovery independently from the slope of cortisol reactivity. Fifth, we specifically focused on amplitude scaling model and multilevel model approaches that were based on the gamma distribution; other model types with intermediate flexibility, or from other base functions, were not examined here and may provide superior results. Sixth, the use of simulated data for the validation of model-based indicators has certain limitations. For example, it is assumed that the stress response was elicited by a discrete stressor (the Trier Social Stress Test). The applicability of the model may not generalize to all real-world data; caution is especially warranted when there is a lack of a discrete stressor, for example due to the use of multiple successive stress paradigms or significant anticipation stress. Moreover, the application of the models to independent empirical data provides limited external validation of our method, since there was no “ground truth” for evaluation of their accuracy. We therefore encourage future validation efforts with empirical data. Seventh, since a significant portion of the STRESS-EU database showed relatively shorter sampling durations (e.g. 40 min), the current study emphasized the assessment of AUC and cortisol peak response indicators over cortisol recovery indicators. Generally, widely recognized indicators of recovery from cortisol responses are lacking and should be a subject of further research. Future research could apply different statistical models that allow more accurate modeling of the recovery phase of the cortisol response, since individual differences herein may be another avenue for the field of psychopathology research (Burke et al., 2005; Dziurkowska and Wesolowski, 2021; Fiksdal et al., 2019).

## 5 Conclusions

Combined data from acute stress test studies requires the use of cortisol stress response indicators that are robust to variability in frequency and duration of saliva sampling (i.e. sampling schedules). Conventional observation-based AUC indicators, however, are inherently correlated with sampling duration, which decreases their accuracy. In this study, we therefore applied two novel model-based indicator approaches that provide inter- and extrapolation of data and as such were expected to improve the stability of indicator estimates (AUCi, AUCg, reactivity and maximum increase) for combined data. Evaluation of model-based indicators with simulated data and their application to independent data support their suitability to combined data, especially with regards to AUC indicators. Extreme sparsity in sampling (e.g. only baseline and 30 min post-stressor onset) is however best avoided in the design of future individual acute stress test studies. Future studies are advised to include at least one additional sample in the recovery phase of the stress response, for instance at 60 min post-stressor onset. Our novel model-based indicator approach can be used by other researchers to harmonize indicators of the cortisol stress response for conducting large-scaled IPD analyses.

## Funding information

This research was supported by the Dutch Organisation for knowledge and innovation in health, healthcare and well-being (ZonMw; 91218038) and the Dutch Research Council (NWO; VI.C.211.106).

## CRediT authorship contribution statement

**Laura de Nooij:** Conceptualization, Data curation, Formal analysis, Methodology, Project administration, Software, Validation, Visualization, Writing – original draft. **Jonathan F. Posthuma:** Conceptualization, Data curation, Formal analysis, Methodology, Project administration, Software, Validation, Visualization, Writing – review & editing. **Robert Miller:** Formal analysis, Methodology, Software, Validation, Writing – review & editing. **Milou S.C. Sep:** Methodology, Writing – review & editing. **Conny Quaedflieg:** Methodology, Writing – review & editing. **Dennis Hernaus:** Methodology, Writing – review & editing. **Christiaan H. Vinkers:** Data curation, Methodology, Resources, Writing – review & editing. **Erno J. Hermans:** Conceptualization, Data curation, Funding acquisition, Methodology, Project administration, Resources, Supervision, Writing – review & editing.

## Declaration of competing interest

All authors declare that they have no conflicts of interest.

## Data Availability

All data for this project (including the multilevel model, which contains participant data) will be made accessible upon publication, although with restrictions; access to these data will require approval from the STRESS-EU consortium (www.stressdatabase.eu).
