# Supplementary material for: Mathematical modeling of the cortisol stress response to develop indicators that are applicable across studies
Source: Neurobiol Stress. 2026 Mar 6;42:100790. doi: 10.1016/j.ynstr.2026.100790 (PMC13000537; doi:10.1016/j.ynstr.2026.100790)
Supplement: Multimedia component 1 [file mmc1.docx]

**Mathematical modeling of the cortisol stress response to develop indicators that are applicable across studies**

**Supplementary Methods**

- 1: Details of statistical modeling
  - Multilevel model with **Figures S1-2**
  - Amplitude scaling model
- 2: Indicators of the cortisol stress response
  - AUCi
  - AUCg
  - Reactivity
  - Maximum increase
- 3: Sets of sampling schedules
  - Representative set
  - High variability set
- 4: Sets of sampling schedules for systematic investigation of expected indicator weaknesses (**Table S1**)
- 5: Simulation of individual variability in sampling timings

**Supplementary Results**

- 1: Bias values in Stress-EU train and test datasets from each train model (**Figure S2**)
- 2: Average cortisol stress response from responder data (with color coding per acute stress test) (**Figure S3**)
- 3: Spearman correlations between “true” indicator values within the simulated dataset (**Figure S4**)
- 4: Descriptive statistics and error values of indicators (**Tables S2-4)**
- 5: Rank-order accuracy of indicators (**Table S5**)
- 4: Individual variability in sampling
  - Summary of findings with **Table S6**
- 5: Correlations between indicators within independent datasets (**Figures S6-10**)

**Supplementary Code**

**References**

**Supplementary Methods 1: Details of statistical modeling**

**Multilevel model**

The multilevel model cortisol stress response to acute stress was estimated according to the formula:

$$cortisol\left( t \right)=A \cdot\frac{\beta^{\alpha}}{\Gamma\left( \alpha\right)}({t-dT)}^{\alpha-1}e^{-\beta x}+b0 +b1 (t-dT)$$

- cortisol = cortisol value in nmol/L
- t = timepoint of saliva sampling in minutes (relative to stressor onset)
- A = amplitude
- $b0$ = population average value at baseline (t=0)
- $\alpha$ = shape parameter from gamma distribution
- $\beta$ = rate parameter from gamma distribution
- $dT$ = parameter to shift the cortisol stress response in time
- *b*1 = parameter to account for the cortisol diurnal rhythm (“slow drift”)

With the saemix package, the estimated multilevel model provides population (sample) and individual (participant) estimates for the participants that that are included in the model fitting procedure. It does not allow direct derivation of individual predictions for new data. Therefore, it was required that data from a new individual was added to the dataset and that the model was refitted; this was repeated for each new participant to extract individual predictions. Here, all parameters except the amplitude parameter were constrained to the values from the previously trained model. Refitting results in slightly varying amplitude parameter and covariate estimates, which means estimations are not completely independent. With the current model training sample size, this problem is expected to be only minor. In the worst case, the individual fit could be affected by overfitting, which would eventually negatively affect indicator estimation. Further development of the saemix package in the future could potentially improve multilevel model estimates. The R syntax for fitting the multilevel model with R package saemix (Comets et al., 2017) is provided in the **Supplementary Code**, see **Box 1**. The initial values for the model correspond to the curve in **Figure S1**.


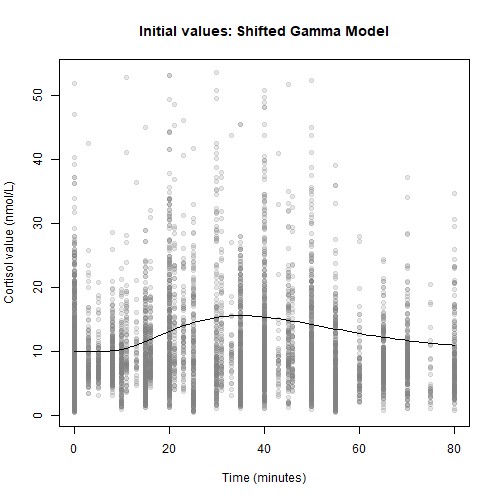


**Figure S1**: Cortisol response curve according to initial values prior to multilevel model fitting.

**
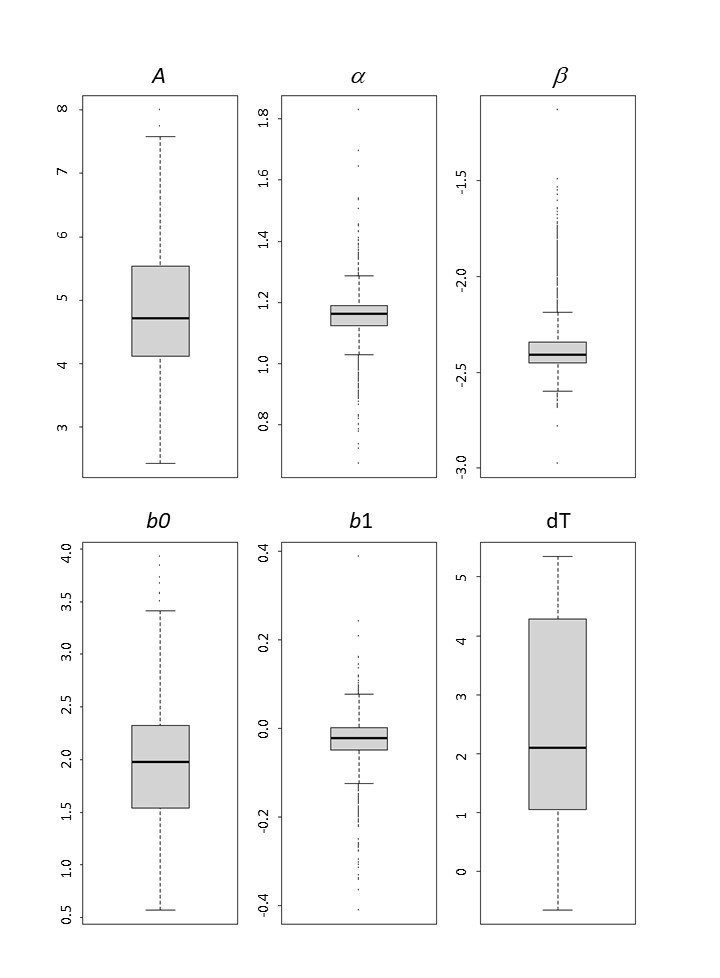
**

**Figure S2**: Individual variability in parameters of the multilevel model (produced with package saemix)

**Amplitude scaling model**

The final population average cortisol response curve to acute stress was estimated according to the formula:

$$cortisol\left( t \right)=A \cdot\frac{\beta^{\alpha}}{\Gamma\left( \alpha\right)}t^{\alpha-1}e^{-\beta x}+b0$$

- cortisol = cortisol value in nmol/L
- t = timepoint of saliva sampling in minutes (relative to stressor onset)
- A = amplitude
- $b0$ = population average value at baseline (t=0)
- $\alpha$ = shape parameter from gamma distribution
- $\beta$ = rate parameter from gamma distribution

The full Stress-EU stress condition responder participant dataset was fitted to this formula, which resulted in the following parameter estimates:

- A = 316.96497
- *b*0 = 8.57258
- $\alpha$ = 4.64952
- $\beta$ = 0.12556

This formula can be implemented using a gamma probability density function:

$$y(x) = A*gamma.pdf(x = x, a= \alpha, scale=1/\beta) + b0$$

With estimated parameters, this results in the following function:

$$y(x) = 316.96497*gamma.pdf(x = x, a= 4.64952, scale=1/0.12556)+8.57258$$

According to the amplitude scaling model, this population average cortisol response curve is fitted to a single individual by adding and estimating an individual amplitude scaling parameter (A_indiv_) to the above formula. This one free parameter should be estimated with a fit to the observations of that individual, so that one can inter- and extrapolate all datapoints of that individual.

**Supplementary Methods 2: Indicators of the cortisol stress response**

**AUC with respect to increase (AUCi)**
Area under the curve minus area below baseline cortisol value (Pruessner et al., 2003).

**AUC with respect to ground (AUCg)**
Area under the curve (Pruessner et al., 2003).

**Reactivity**
Cortisol value at a peak timepoint minus cortisol value at baseline (Khoury et al., 2015). In this study, we use the timepoint at 30 minutes relative to stressor onset as peak timepoint. If no cortisol value is available for this timepoint, the cortisol value from the timepoint closest in time is used. In case two timepoints are equally close in time (e.g. 20, 40), the cortisol values from both timepoints are averaged.

**Maximum increase**
Maximum value (0 to 60 minutes relative to stressor onset) minus minimum value (0 to 60 minutes relative to stressor onset) (Miller et al., 2018). The minimum value can be at baseline or in recovery, so that this indicator represents the maximum dynamic range of the system.

**Supplementary Methods 3: Sampling schedules for aggregated data analyses**

**Representative set**
Twelve sampling schedules:

RS1 [0, 30]

RS2 [0, 20, 40]

RS3 [0, 10, 20, 30, 40]

RS4 [0, 30, 60]

RS5 [0, 20, 40, 60]

RS6 [0, 20, 30, 40, 60]

RS7 [0, 20, 40, 60, 80]

RS8 [0, 20, 30, 40, 60, 80]

RS9 [0, 10, 20, 30, 40, 50, 60, 70, 80]

RS10 [0, 15, 35, 55]

RS11 [0, 15, 20, 25, 35, 55]

RS12 [0, 15, 20, 25, 35, 55, 70]

**High variability set**
Five sampling schedules:

HV1 [0, 30]

HV2 [0, 10, 20, 30, 40]

HV3 [0, 15, 35, 55]

HV4 [0, 15, 20, 25, 35, 55, 70]

HV5 [0, 20, 40, 60, 80]

**Supplementary Methods 4: Sets of sampling schedules for systematic investigation of expected indicator weaknesses**

**Table S1:** Sets of sampling schedules that were used to systematically investigate to what extent expected weaknesses of each indicator pose a threat to the stability of the indicator estimates (across different sampling schedules) and how this may differ between conventional observation-based and model-based methods. Within each set, sampling schedules differ only on one specific factor.

| Factor | Indicator(s) | Set(s) of sampling schedules  Timepoints in minutes relative to stressor onset | | | |
| --- | --- | --- | --- | --- | --- |
| Duration | AUCi, AUCg | 1. 0,10,20,30,40 0,10,20,30,40,50 0,10,20,30,40,50,60 0,10,20,30,40,50,60,70 0,10,20,30,40,50,60,70,80 | | | |
|  | Maximum increase | 1. 0,10,20,30,40   0,10,20,30,40,50 0,10,20,30,40,50,60 | | | |
| Frequency | AUCi, AUCg,  Maximum increase | 1. 0,40,80   0,20,40,60,80  0,10,20,30,40,50,60,70,80 | | 1. 0,30,60   0,15,30,45,60  0,10,20,30,40,50,60 | |
| Peak timepoint | Reactivity**,** Maximum increase | 1. 0,30*   0,35*  0,40*  0,20*,40*  0,25*,35* | 1. 0,30*,60   0,35*,60  0,40*,60  0,20*,40*,60  0,25*,35*,60 | | 1. 0,35*,80   0,40*,80  0,25*,35*,80 |

AUCg = Area Under the Curve with respect to Ground, AUCi = Area Under the Curve with respect to Increase.

**Superscripts:** * indicates peak timepoint(s); in case of two peak timepoints, the average cortisol value is used.

**Supplementary Methods 5: Simulation of individual variability in sampling**

To simulate logistically challenging studies that face delays in their procedures, leading to individual variation in sampling timings, we added random delays to the sampling schedules for each participant. This was achieved with random sampling from gaussian distributions (M = 0) that were truncated from the mean, i.e. only positive values. To avoid extreme values that go beyond the end of the simulated curve, the gaussian distribution was also truncated at +2SD. With these methods we simulated two sets of sampling timings.

The first set was based on sampling schedule [0, 20 ,40, 60, 80]. To mimic a study that may face delays in its procedures from stressor onset, but contains a sufficient gap until the last measurement in recovery, delays were only added to timepoints [20, 40, 60]. For each timepoint, the delay was added to that timepoint and all subsequent timepoints. All delays were sampled from a truncated gaussian distribution with SD=3. This process was ten times repeated, leading to ten datasets with medium individual variability in sampling schedules according to M(SD): [0, 21.7(1.5), 43.9(2.2), 66.1(2.6), 80].

The second set was based on sampling schedule [0, 25, 50] (in minutes relative to stressor onset). The first delay was sampled from a truncated gaussian distribution with SD=10 and added to both timepoints [25,50]. Then a second delay sampled from a truncated gaussian distribution with SD=5 added was added only to timepoint [50]. This process was ten times repeated, leading to ten datasets with high individual variability in sampling schedules according to M(SD): [0, 31.8(5.0), 60.4(5.6)].

**Supplementary Results 1: Bias values in Stress-EU train and test datasets from each train model**

**
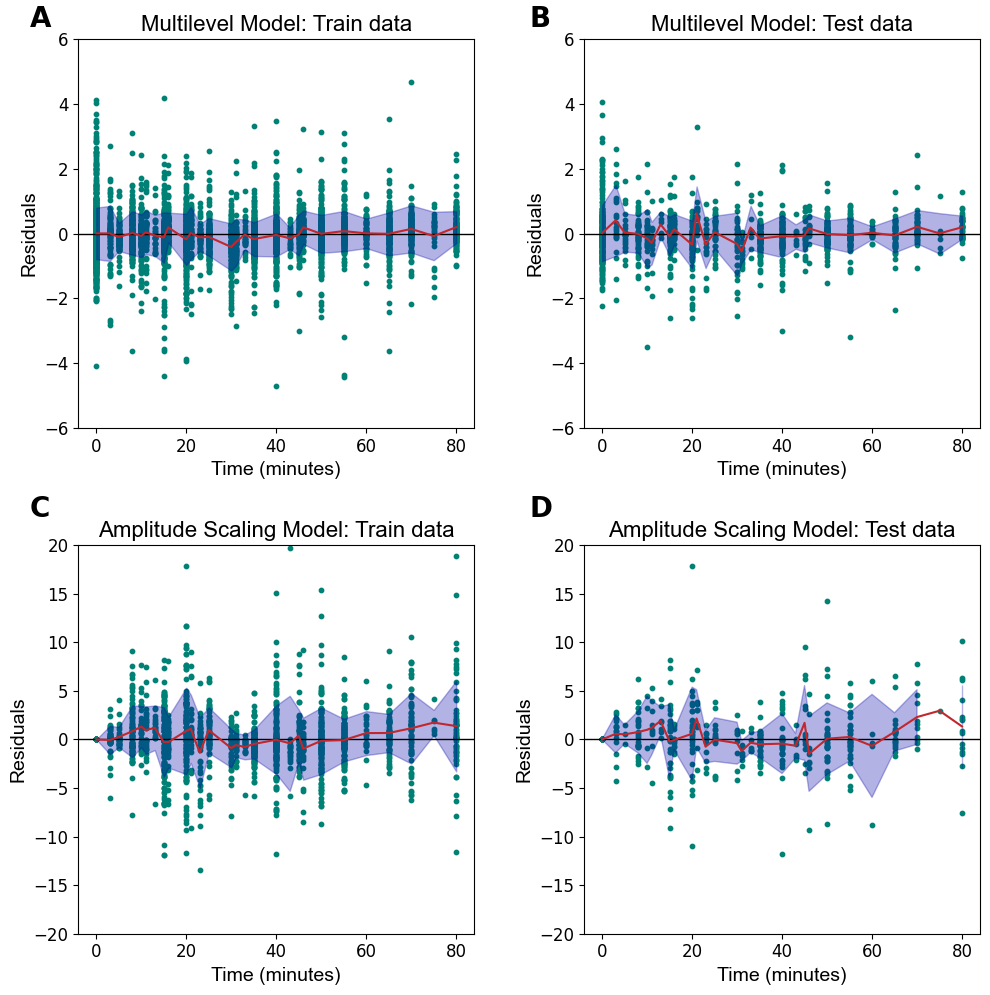
**

**Figure S3:** Bias values (i.e., mean residuals) in STRESS-EU train and test datasets from each train model. Points show each observation in the dataset, whereas the solid line and shading reflect the mean and standard deviation.
**Note**: Different scaling of y-axes indicates that the multilevel model produces significantly lower error values.

**Supplementary Results 2: Average cortisol stress response from responder data (with color coding per acute stress test)**

**
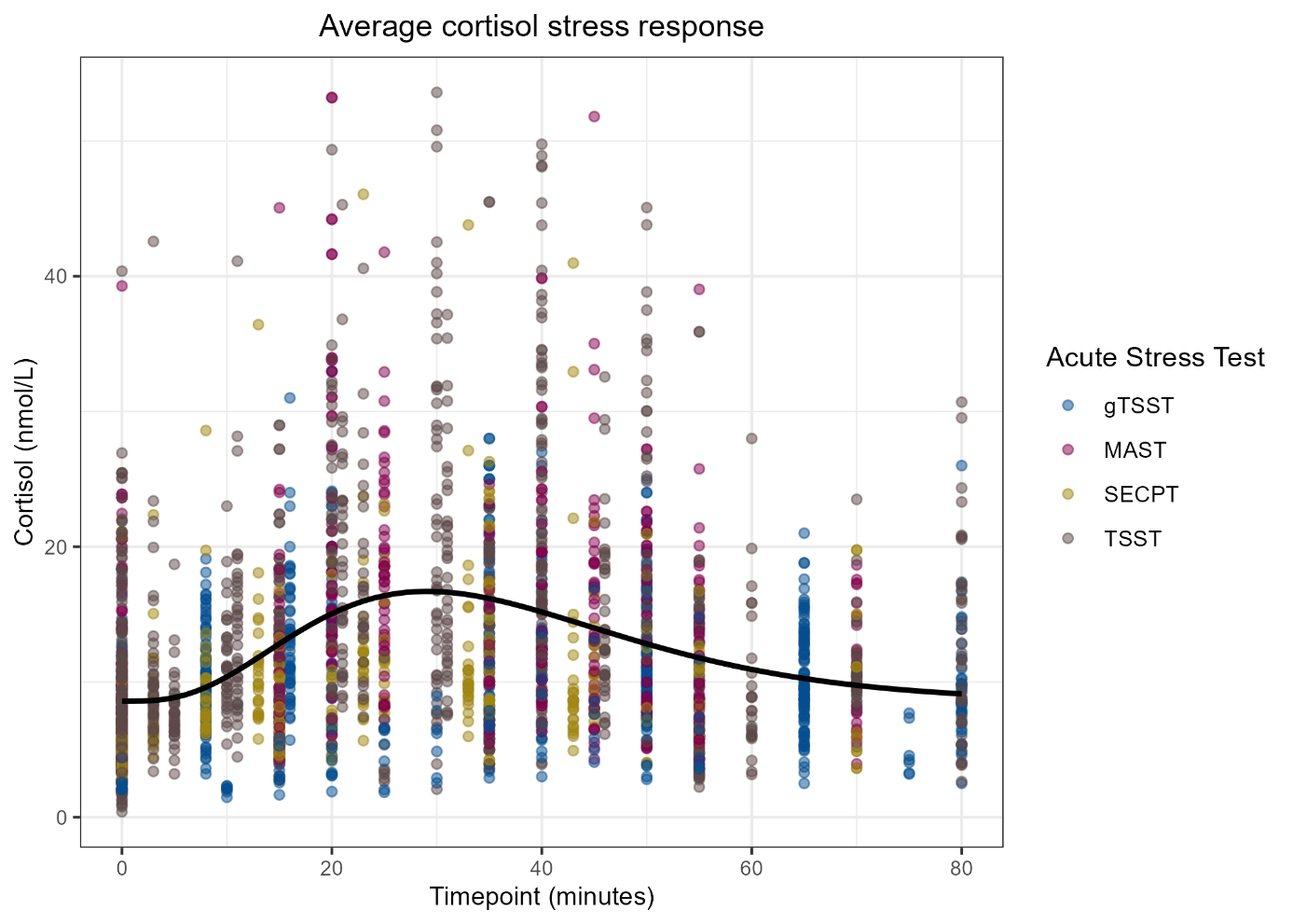
**

**Figure S4:** The population average cortisol response curve (solid line) which was fitted on observations (datapoints) from stress condition responder participants. This figure shows different colors in datapoints to indicate different acute stress tests.

**Supplementary Results 3: Spearman correlations between “true” indicator values within the simulated dataset.**


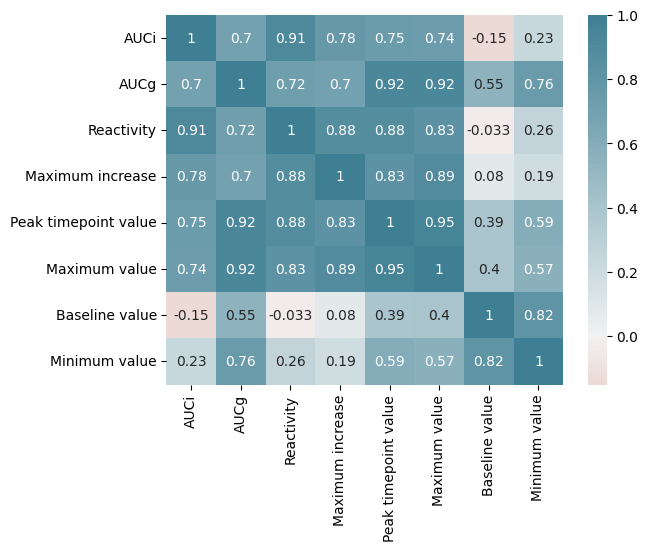


**Figure S5:** Spearman correlations between “true” indicator values within the simulated dataset.

**Supplementary Results 4: Descriptive statistics and error values of indicators**

**Table S2**: Descriptive statistics M(SD) of indicators according to three methods for twelve different sampling schedules (RS1-RS12) that were applied to the simulated dataset.

| **Indicator** | **Method** | **Sampling schedule** | | | | | | | | | | | | **M_M_**  **(SD_M_)** |
| --- | --- | --- | --- | --- | --- | --- | --- | --- | --- | --- | --- | --- | --- | --- |
|  |  | RS1 | RS2 | RS3 | RS4 | RS5 | RS6 | RS7 | RS8 | RS9 | RS10 | RS11 | RS12 |  |
| AUCi | OBS | 74.6  (82.8) | 138.1  (150.1) | 136.0 (149.0) | 165.3 (193.0) | 181.4 (206.6) | 188.5 (213.2) | 195.9 (237.9) | 203.0 (244.3) | 190.0 (233.7) | 165.2 (188.5) | 182.6 (203.8) | 197.8 (230.9) | 168.2  (36.7) |
|  | MM | 210.1  (226.9) | 184.7 (237.6) | 181.3 (233.4) | 157.0 (213.2) | 162.8 (226.9) | 161.2 (225.2) | 166.1 (221.8) | 165.3 (219.4) | 163.8 (218.1) | 160.9 (228.1) | 161.1 (226.6) | 165.1 (221.4) | 169.9  (15.1) |
|  | ASM | 190.5 (211.6) | 202.2 (224.0) | 199.2 (218.9) | 186.7 (213.0) | 198.4 (224.3) | 195.1 (217.0) | 198.5 (225.3) | 195.2 (217.5) | 191.0 (218.0) | 189.3 (218.8) | 212.6 (231.5) | 212.3 (232.0) | 197.3  (8.3) |
| AUCg | OBS | 205.4  (99.6) | 312.4 (165.1) | 310.4 (162.9) | 426.8 (217.2) | 442.9 (228.2) | 450.0 (234.7) | 544.7 (264.8) | 551.7 (271.2) | 538.7 (259.6) | 404.9 (207.9) | 422.4 (222.5) | 503.0 (254.1) | 426.1  (106.5) |
|  | MM | 590.1  (256.9) | 571.4 (270.7) | 562.9 (266.0) | 541.9 (248.5) | 548.3 (260.6) | 546.8 (258.5) | 550.2 (257.3) | 548.9  (255.3) | 543.1  (257.0) | 546.7 (261.2) | 546.8 (259.2) | 549.7 (255.8) | 553.2  (14.1) |
|  | ASM | 539.3  (258.0) | 550.9 (264.5) | 547.9 (261.0) | 535.4 (255.4) | 547.2 (262.1) | 543.9 (258.7) | 547.2 (262.1) | 543.9 (258.7) | 539.8 (256.2) | 538.0 (256.8) | 561.4 (270.4) | 561.0 (270.0) | 546.3  (8.3) |
| Reactivity | OBS | 4.97  (5.53) | 4.27  (4.72) | 4.97  (5.53) | 4.97  (5.53) | 4.27  (4.72) | 4.97  (5.53) | 4.27  (4.72) | 4.97  (5.53) | 4.97  (5.53) | 4.11  (4.94) | 4.79  (5.30) | 4.79  (5.30) | 4.70  (0.35) |
|  | MM | 4.60  (5.31) | 4.24  (5.22) | 4.48  (5.32) | 4.42  (5.38) | 4.46  (5.38) | 4.50  (5.38) | 4.47  (5.40) | 4.53  (5.37) | 4.56  (5.30) | 4.32  (5.39) | 4.52  (5.34) | 4.57  (5.33) | 4.47  (0.10) |
|  | ASM | 4.97  (5.53) | 5.28  (5.85) | 5.20  (5.72) | 4.87  (5.56) | 5.18  (5.86) | 5.09  (5.67) | 5.18  (5.88) | 5.10  (5.68) | 4.99  (5.69) | 4.94  (5.71) | 5.55  (6.05) | 5.54  (6.06) | 5.16  (0.22) |
| Maximum increase | OBS | 5.21  (5.30) | 6.06 (5.45) | 6.68 (5.52) | 5.82 (5.10) | 6.52  (5.34) | 6.93  (5.51) | 6.93  (5.32) | 7.33  (5.50) | 7.65  (5.40) | 5.97  (5.86) | 7.07  (5.57) | 7.43  (5.55) | 6.63  (0.74) |
|  | MM | 5.43  (5.18) | 6.46  (5.62) | 6.66  (5.55) | 5.63  (5.40) | 6.25  (5.84) | 6.29  (5.79) | 6.12  (5.87) | 6.13  (5.81) | 6.12  (5.71) | 6.06  (5.67) | 6.34  (5.69) | 6.14  (5.67) | 6.14  (0.33) |
|  | ASM | 5.22  (5.31) | 5.55  (5.61) | 5.44  (5.51) | 5.19  (5.29) | 5.50  (5.57) | 5.35  (5.44) | 5.52  (5.59) | 5.36  (5.45) | 5.32  (5.41) | 5.30  (5.40) | 5.77  (5.86) | 5.78  (5.86) | 5.44  (0.19) |

**Abbreviations:** AUCg = Area Under the Curve with respect to Ground, AUCi = Area Under the Curve with respect to Increase, ASM = amplitude scaling model, MM = multilevel model, OBS = conventional observation-based method.

**Table S3**: Mean Absolute Error (MAE) values indicating mean absolute differences between true indicator values and estimated indicator values according to three methods for twelve different sampling schedules (RS1-RS12) that were applied to the simulated dataset.

| **Indicator** | **Method** | **Sampling schedule** | | | | | | | | | | | | **M_MAE_** | **SD_MAE_** |
| --- | --- | --- | --- | --- | --- | --- | --- | --- | --- | --- | --- | --- | --- | --- | --- |
|  |  | RS1 | RS2 | RS3 | RS4 | RS5 | RS6 | RS7 | RS8 | RS9 | RS10 | RS11 | RS12 |  |  |
| AUCi | OBS | 133.3 | 81.4 | 82.4 | 57.4 | 50.2 | 48.3 | 52.2 | 52.9 | 50.8 | 58.0 | 52.4 | 48.3 | 64.0 | 24.8 |
|  | MM | 79.9 | 71.5 | 74.6 | 65.9 | 57.7 | 57.7 | 56.3 | 55.8 | 55.4 | 61.9 | 60.3 | 56.6 | 62.8 | 8.3 |
|  | ASM | 63.5 | 58.8 | 57.3 | 55.7 | 52.2 | 52.2 | 51.6 | 51.6 | 47.2 | 51.5 | 63.5 | 61.9 | 55.6 | 5.4 |
| AUCg | OBS | 332.6 | 225.6 | 227.6 | 111.9 | 95.5 | 88.4 | 28.0 | 29.0 | 18.1 | 133.2 | 115.9 | 40.9 | 120.6 | 96.9 |
|  | MM | 88.8 | 71.1 | 68.9 | 47.9 | 35.9 | 35.2 | 30.2 | 28.5 | 18.8 | 42.0 | 39.6 | 28.9 | 44.6 | 20.9 |
|  | ASM | 64.5 | 54.6 | 53.6 | 52.2 | 43.8 | 47.8 | 41.7 | 46.4 | 36.2 | 42.2 | 59.4 | 56.8 | 49.9 | 8.3 |
| Reactivity | OBS | 0.90 | 1.32 | 0.90 | 0.90 | 1.32 | 0.90 | 1.32 | 0.90 | 0.90 | 1.42 | 0.88 | 0.88 | 1.05 | 0.22 |
|  | MM | 0.87 | 1.32 | 0.93 | 0.97 | 1.26 | 0.93 | 1.29 | 0.95 | 0.94 | 1.21 | 0.95 | 0.93 | 1.05 | 0.17 |
|  | ASM | 0.90 | 1.31 | 1.04 | 1.06 | 1.38 | 1.02 | 1.42 | 1.04 | 1.22 | 1.34 | 1.30 | 1.32 | 1.20 | 0.17 |
| Maximum increase | CONV | 2.18 | 1.45 | 1.06 | 1.69 | 1.16 | 0.92 | 1.13 | 0.99 | 1.02 | 1.54 | 0.87 | 0.93 | 1.25 | 0.4 |
|  | MM | 2.13 | 1.50 | 1.33 | 1.84 | 1.33 | 1.20 | 1.42 | 1.30 | 1.24 | 1.51 | 1.12 | 1.25 | 1.43 | 0.29 |
|  | ASM | 2.18 | 1.97 | 1.99 | 2.29 | 2.10 | 2.08 | 2.11 | 2.09 | 2.22 | 2.27 | 1.75 | 1.77 | 2.07 | 0.18 |

**Abbreviations:** AUCg = Area Under the Curve with respect to Ground, AUCi = Area Under the Curve with respect to Increase, ASM = amplitude scaling model, MM = multilevel model, OBS = conventional observation-based method.

**Table S4**: Bias values indicating mean difference values (true indicator – estimated indicator) according to three methods for twelve different sampling schedules (RS1-RS12) that were applied to the simulated dataset.

| **Indicator** | **Method** | **Sampling schedule** | | | | | | | | | | | | **M_bias_** | **SD_bias_** |
| --- | --- | --- | --- | --- | --- | --- | --- | --- | --- | --- | --- | --- | --- | --- | --- |
|  |  | RS1 | RS2 | RS3 | RS4 | RS5 | RS6 | RS7 | RS8 | RS9 | RS10 | RS11 | RS12 |  |  |
| AUCi | OBS | 114.4 | 51.0 | 53.0 | 23.8 | 7.7 | 0.6 | −6.9 | −13.9 | −0.9 | 23.9 | 6.5 | −8.7 | 20.9 | 36.7 |
|  | MM | −21.0 | 4.3 | 7.7 | 32.1 | 26.3 | 27.8 | 22.9 | 23.8 | 25.3 | 28.2 | 28.0 | 23.9 | 19.1 | 15.1 |
|  | ASM | −1.4 | −13.1 | −10.1 | 2.4 | −9.4 | −6.1 | −9.4 | −6.1 | −2.0 | −0.2 | −23.6 | −23.2 | −8.5 | 8.3 |
| AUCg | OBS | 332.6 | 225.6 | 227.6 | 111.2 | 95.1 | 88.0 | −6.7 | −13.7 | −0.7 | 133.1 | 115.7 | 35.1 | 111.9 | 106.5 |
|  | MM | −52.0 | −33.4 | −24.9 | −3.9 | −10.2 | −8.8 | −12.1 | −10.9 | −5.1 | −8.6 | −8.6 | −11.6 | −15.9 | 14.1 |
|  | ASM | −1.2 | −12.9 | −9.9 | 2.6 | −9.2 | −5.8 | −9.2 | −5.9 | −1.8 | 0.0 | −23.3 | −23.0 | −8.3 | 8.3 |
| Reactivity | OBS | −0.01 | 0.69 | −0.01 | −0.01 | 0.69 | −0.01 | 0.69 | −0.01 | −0.01 | 0.85 | 0.17 | 0.17 | 0.26 | 0.35 |
|  | MM | 0.36 | 0.72 | 0.48 | 0.54 | 0.50 | 0.46 | 0.49 | 0.43 | 0.40 | 0.64 | 0.44 | 0.39 | 0.49 | 0.10 |
|  | ASM | −0.01 | −0.32 | −0.24 | 0.09 | −0.22 | −0.13 | −0.22 | −0.14 | −0.03 | 0.02 | −0.59 | −0.58 | −0.20 | 0.22 |
| Maximum increase | OBS | 2.00 | 1.15 | 0.52 | 1.39 | 0.69 | 0.27 | 0.27 | −0.12 | −0.44 | 1.23 | 0.14 | −0.22 | 0.57 | 0.74 |
|  | MM | 1.78 | 0.75 | 0.55 | 1.58 | 0.96 | 0.91 | 1.09 | 1.08 | 1.08 | 1.14 | 0.87 | 1.06 | 1.07 | 0.33 |
|  | ASM | 1.99 | 1.66 | 1.77 | 2.02 | 1.70 | 1.86 | 1.68 | 1.85 | 1.89 | 1.90 | 1.43 | 1.43 | 1.77 | 0.19 |

**Abbreviations:** AUCg = Area Under the Curve with respect to Ground, AUCi = Area Under the Curve with respect to Increase, ASM = amplitude scaling model, MM = multilevel model, OBS = conventional observation-based method.

**Supplementary Results 5: Rank-order accuracy of indicators**

**Table S5**: Spearman *r* correlation coefficients of correlations between observed and true indicator values per sampling schedule (*r_s_*_, single_) according to three methods for twelve different sampling schedules (RS1-RS12) that were applied to the simulated dataset.

| **Indicator** | **Method** | **Sampling schedule** | | | | | | | | | | | | **M*r_s_*_,single_^T^** |
| --- | --- | --- | --- | --- | --- | --- | --- | --- | --- | --- | --- | --- | --- | --- |
|  |  | RS1^Z^ | RS2^Z^ | RS3^Z^ | RS4^Z^ | RS5^Z^ | RS6^Z^ | RS7^Z^ | RS8^Z^ | RS9^Z^ | RS10^Z^ | RS11^Z^ | RS12^Z^ |  |
| AUCi | OBS | 0.864 | 0.872* | 0.871* | **0.904*** | 0.916 | **0.921*** | **0.920*** | **0.926*** | 0.922 | 0.906 | **0.912*** | **0.926*** | 0.905 |
|  | MM | *0.822** | *0.854** | *0.829** | *0.880** | *0.905** | 0.908 | *0.900** | *0.904** | *0.905** | *0.890** | 0.896 | 0.901 | *0.883** |
|  | ASM | 0.864 | **0.893*** | **0.896*** | 0.895* | 0.912 | 0.911 | 0.915* | 0.913* | 0.924 | 0.910 | 0.894 | 0.899 | 0.902 |
| AUCg | OBS | 0.918 | 0.932* | 0.939* | **0.959*** | 0.971 | 0.974* | **0.983*** | **0.985*** | 0.992 | **0.965*** | **0.967*** | **0.984*** | **0.964*** |
|  | MM | *0.880** | *0.910** | *0.894** | 0.948* | 0.972 | 0.972* | 0.980* | 0.983* | 0.992 | *0.957** | 0.961***** | 0.978* | 0.952 |
|  | ASM | 0.915 | **0.943*** | **0.944*** | *0.941** | *0.961** | *0.953** | *0.965** | *0.956** | *0.971** | 0.961 | *0.944** | *0.949** | 0.950 |
| Reactivity | OBS | 0.944 | 0.907 | 0.944 | **0.944*** | **0.907*** | 0.944 | **0.907*** | **0.944*** | **0.944*** | 0.902 | **0.944*** | **0.944*** | **0.932*** |
|  | MM | **0.947*** | *0.887** | 0.942 | 0.940* | 0.899 | 0.942 | 0.895 | 0.939 | 0.941 | **0.910*** | 0.929 | 0.940* | 0.927 |
|  | ASM | 0.944 | 0.907 | *0.935** | *0.931** | *0.898** | *0.935** | *0.895** | *0.933** | *0.915** | 0.901 | *0.925** | *0.923** | 0.920 |
| Maximum increase | OBS | 0.824 | **0.877*** | **0.896*** | 0.857 | 0.900* | 0.918* | 0.884* | 0.903* | 0.909* | 0.877 | 0.922* | 0.915* | 0.890 |
|  | MM | *0.791** | 0.833 | 0.843 | 0.853 | **0.906*** | **0.924*** | **0.904*** | **0.920*** | **0.935*** | 0.881 | **0.928*** | **0.922*** | 0.887 |
|  | ASM | 0.824 | 0.827 | 0.843 | *0.790** | *0.799** | *0.827** | *0.793** | *0.824** | *0.793** | *0.778** | *0.854** | *0.848** | *0.817** |

**Abbreviations:** ASM = amplitude scaling model, AUCg = Area Under the Curve with respect to Ground, AUCi = Area Under the Curve with respect to Increase, MM = multilevel model, OBS = conventional observation-based method.
**Superscripts:** ^Z^ Pairwise Z-tests to compare Z-scores, ^T^ Paired t-tests to compare vectors of Spearman correlation coefficients, * Statistically significant (*p* < .05) difference between methods.
**Note:** the values indicate mean Spearman *r* correlation coefficient of “true” with estimated values from application of single schedules to data (M*r*_s,indiv_). Based on the test results, **bold** faced numbers present the most favourable method, and *italics* the least favourable method.

**Supplementary Results 6: Individual variability in sampling**

**Summary of findings**

For simulated data with medium individual variability, conventional observation-based AUCi and AUCg (calculated with the average sampling timepoints across the sample) were most accurate (all *p* < .001). Regarding maximum increase and reactivity, we did see some accuracy improvement with multilevel model-based indicators (reactivity M*r_s_*_,indiv_(9998) = 0.900, maximum increase M*r_s_*_,indiv_(9998) = 0.897) as compared to conventional observation-based indicators (reactivity M*r_s_*_,indiv_(9998) = 0.874, maximum increase M*r_s_*_,indiv_(9998) = 0.878; both *p* < .001). With our high individual variability sample, amplitude scaling model-based AUCi performed most accurately (all *p* < .001), but the other three amplitude scaling model-based indicators were least accurate (all *p <* .001). Multilevel model-based AUCg [M*r_s_*_,indiv_ (9998) = 0.950] then minimally outperforms conventional observation-based AUCg [M*r_s_*_,indiv_(9998) = 0.941;  *p*  < .001]; for reactivity and maximum increase they were equally most accurate.

**Table S6**: Rank-order accuracy of indicators derived from simulated data according to three methods in case of individual variability in sampling times.

| Indicator | Data | Method | | |
| --- | --- | --- | --- | --- |
|  |  | OBS | MM | ASM |
| AUCi | MIV | **0.919*** | *0.901** | 0.913 |
|  | HIV | 0.881 | 0.874 | **0.884*** |
| AUCg | MIV | **0.980*** | 0.976 | *0.963** |
|  | HIV | 0.941 | **0.946*** | *0.937** |
| Reactivity | MIV | *0.874** | **0.900*** | 0.895 |
|  | HIV | 0.897 | 0.897 | *0.892** |
| Maximum increase | MIV | 0.878 | **0.897*** | *0.797** |
|  | HIV | 0.808 | 0.812 | *0.749** |

**Abbreviations:** AUCg = Area Under the Curve with respect to Ground, AUCi = Area Under the Curve with respect to Increase, ASM = amplitude scaling model, HIV = high individual variability, MIV = medium individual variability, MM = multilevel model, OBS = conventional observation-based method.
**Superscripts:** Asterisks denote statistically significant results (*p <* .05) from pairwise permutation tests that compared vectors of Spearman correlation coefficients between “true” and estimated indicator values across ten datasets with individual sampling times. These indicate a difference in rank-order accuracy between methods.
**Note:** The values indicate mean Spearman correlation coefficients between “true” and estimated indicator values across ten datasets with individual sampling times (M*r_s_*_,indiv_). Based on statistical testing, **bold** faced numbers present the most favourable method, and *italics* the least favourable method.

**Supplementary Results 7: Correlations between indicators within independent datasets**

**Figure S6:** Spearman correlation coefficients of correlations between indicator values for stress condition procedures in the first independent dataset (Tutunji et al.). Only statistically significant (*p <* .05) coefficients are displayed in this figure.

*
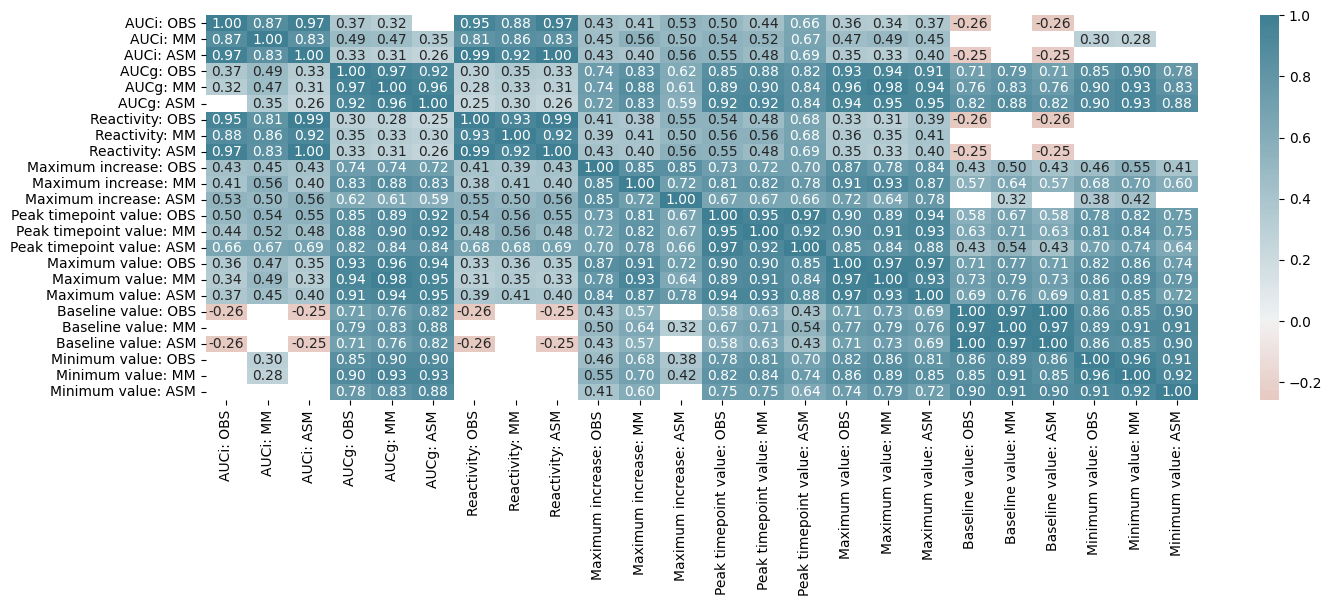
*

AUCg = Area Under the Curve with respect to Ground, AUCi = Area Under the Curve with respect to Increase, ASM = amplitude scaling model, MM = multilevel model, OBS = conventional observation-based method.

**Figure S7:** Spearman correlation coefficients of correlations between indicator values for control condition procedures in the first independent dataset (Tutunji et al.). Only statistically significant (*p <* .05) coefficients are displayed in this figure.

*
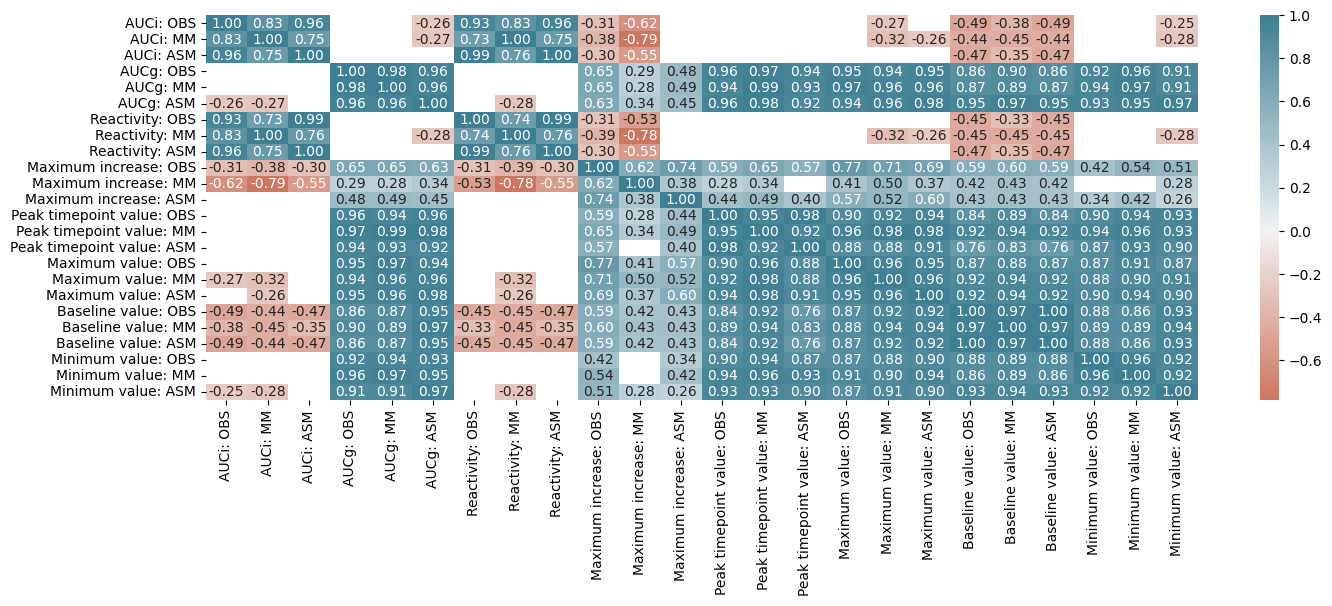
*

AUCg = Area Under the Curve with respect to Ground, AUCi = Area Under the Curve with respect to Increase, ASM = amplitude scaling model, MM = multilevel model, OBS = conventional observation-based method.

**Figure S8:** Spearman correlations coefficient of correlations between indicator values for the stress condition group in the second independent dataset (Voulgaropoulou et al.). Only statistically significant (*p <* .05) coefficients are displayed in this figure.

*
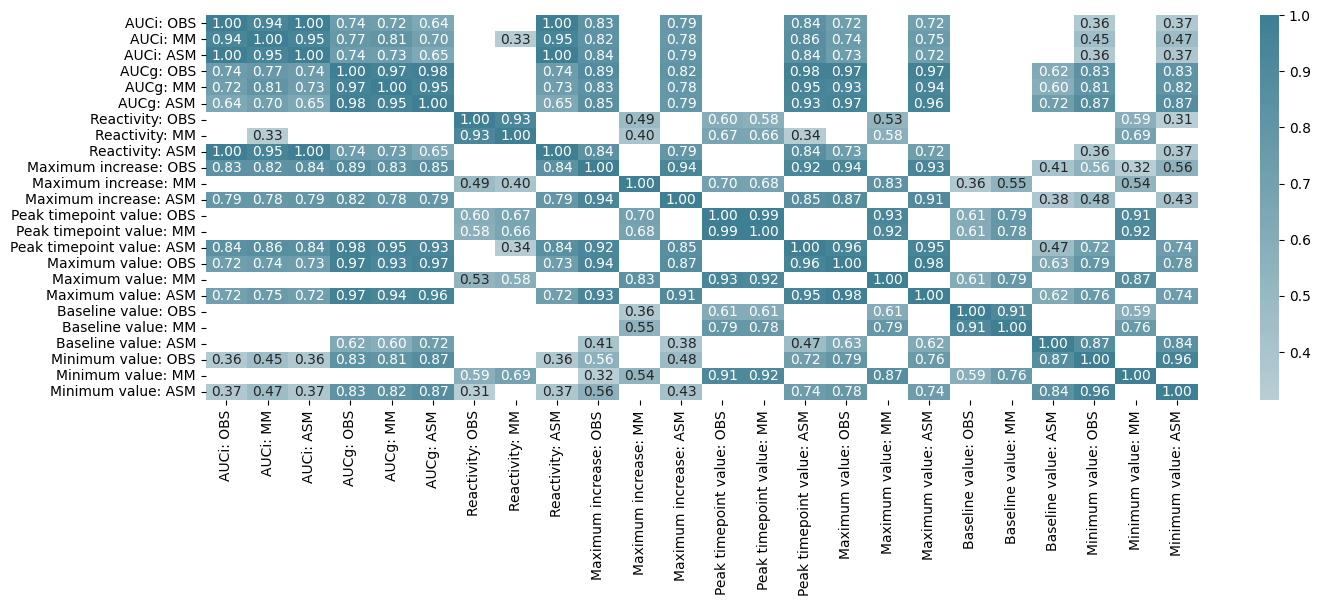
*

AUCg = Area Under the Curve with respect to Ground, AUCi = Area Under the Curve with respect to Increase, ASM = amplitude scaling model, MM = multilevel model, OBS = conventional observation-based method.

**Figure S9:** Spearman correlations coefficients of correlations between indicator values for the control condition group in the second independent dataset (Voulgaropoulou et al.). Only statistically significant (*p <* .05) coefficients are displayed in this figure.

**
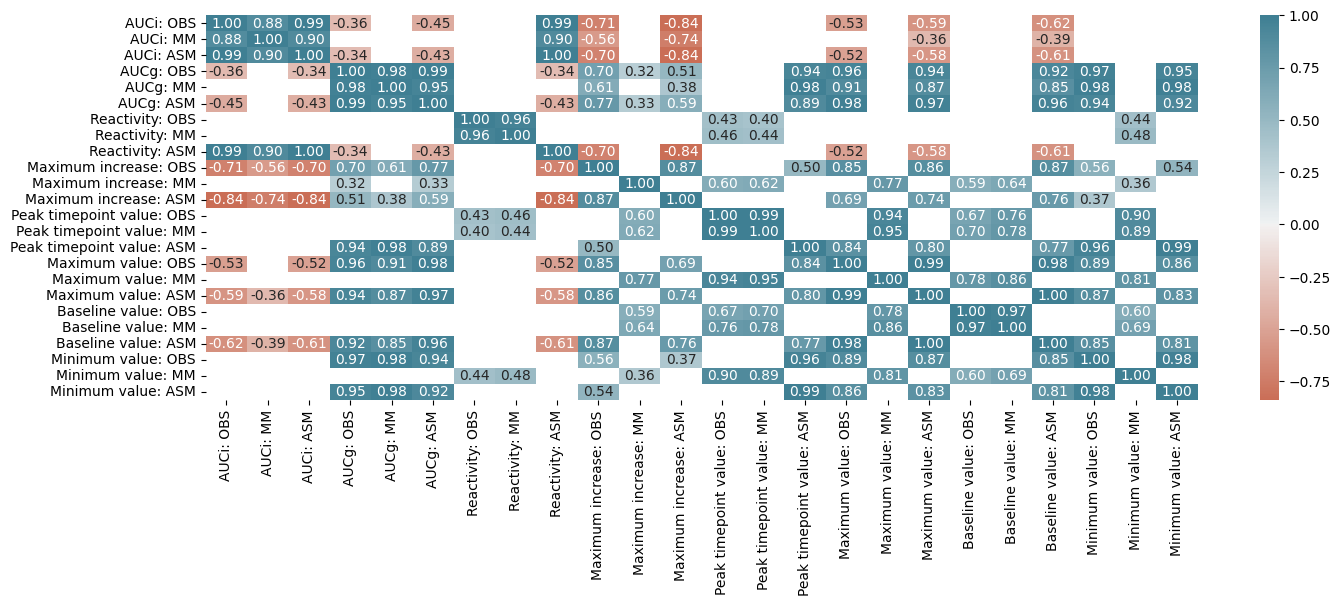
**

AUCg = Area Under the Curve with respect to Ground, AUCi = Area Under the Curve with respect to Increase, ASM = amplitude scaling model, MM = multilevel model, OBS = conventional observation-based method.

**Figure S10:** Spearman correlation coefficients of correlations between indicator values for all procedures in aggregated independent dataset. Only statistically significant (*p <* .05) coefficients are shown in this figure.

*
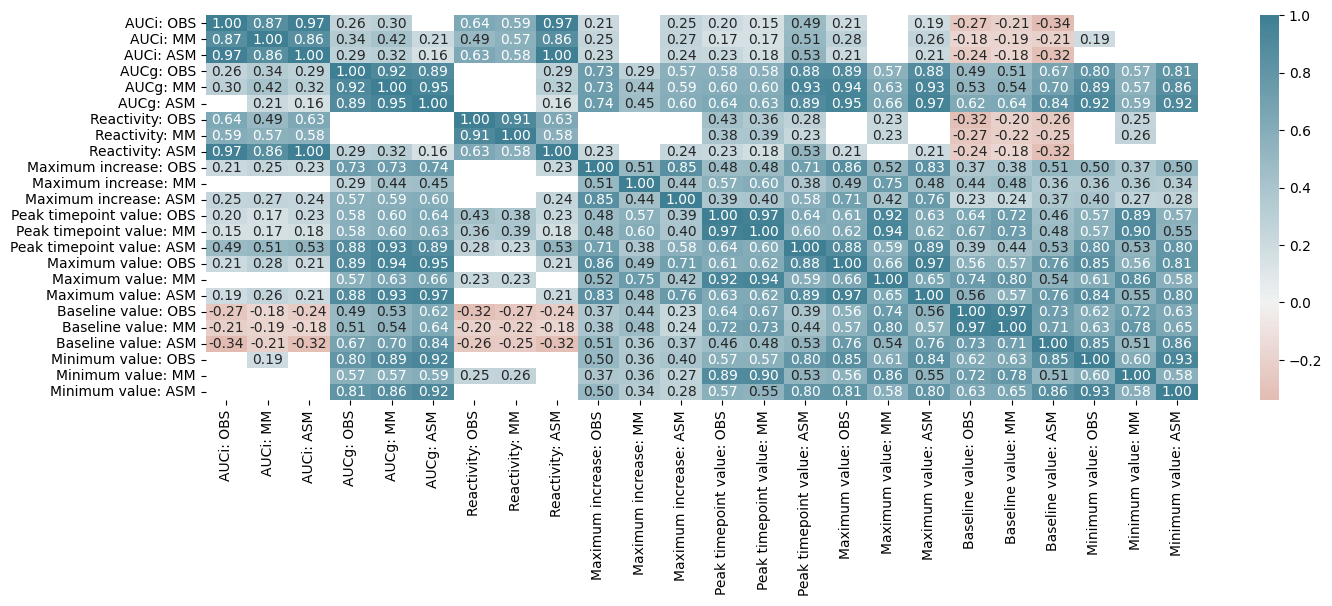
*

AUCg = Area Under the Curve with respect to Ground, AUCi = Area Under the Curve with respect to Increase, ASM = amplitude scaling model, MM = multilevel model, OBS = conventional observation-based method.

**Supplementary code**

**Box 1.** R syntax for multilevel model

library(saemix)
d <- read.csv("data.csv")

***### saemix data***
ds <- saemixData(d,

                 name.group=c("ID"),

                 name.predictors=c("Cortisol_timepoint"),

                 name.response=c("Cortisol_value"),

                 name.covariates=c("gender","dummy_1","dummy_2","dummy_3","dummy_4"),

                 units=list(x="min", y="nM", covariates=c("-","-","-","-","-","-")),

                 name.X="Cortisol_timepoint"

                 )

***### specify structural model function***

mod_gamma <- function(psi, id, xidep){

 ***# predictor(s)***

  time_postStress <- xidep[,1]

***# parameter(s)***

  amplitude <- psi[id,1]

  alpha <- psi[id,2]

  beta <- psi[id,3]

  base_b0 <- psi[id,4]

  base_b1 <- psi[id,5]

  dT <- psi[id,6]

***# prediction for the shifted gamma model (rate parameterization)***  ypred <- amplitude * dgamma(x = time_postStress - dT, shape = alpha, rate = beta) +
 base_b0 + base_b1*time_postStress
  return(ypred)
}

***### set initial parameter values (see Figure S1)***

inits <- c(250, 4, .1, 10, 0, 5) #initial values

times <- seq(min(d$Cortisol_timepoint),

             max(d$Cortisol_timepoint),

             by = 1)

psi <- matrix(inits, ncol=length(inits))

xidep <- matrix(times, ncol=1)

***### specify ful hierarchical model***

mod <- saemixModel(model = mod_gamma, ***#pass structural model***

                   modeltype = "structural",

                   description = "Gamma model - full data",

***# pass initial values***

                   psi0 = matrix(inits, ncol=length(inits), byrow=TRUE,

                   dimnames=list(NULL,

                      c("amplitude","alpha","beta","base_b0","base_b1","dT")

                      )

                  ),

*(****Box 1****. continued)*

***# 5 log-normal parameters + 1 normal parameter***

                   transform.par = c(1,1,1,1,0,1),

***# covariates***

                   covariate.model = matrix(c(1,0,0,1,0,0,

                                              1,0,0,1,1,1,

                                              0,0,0,0,0,0,

                                              0,0,1,1,0,0,

                                              0,0,0,0,0,0), ncol=length(inits),
 byrow=TRUE),

***# 6 parameters freely estimated***

                   fixed.estim = c(1,1,1,1,1,1),

**# diagonal parameter covariance matrix, diagnonal indicates individual
 # variation of parameters, off-diagonal indicates covariation**

                   covariance.model = matrix(c(1,0,0,1,0,0,

                                               0,1,0,0,0,0,

                                               0,0,1,0,0,0,

                                               1,0,0,1,1,0,

                                               0,0,0,1,1,0,

                                               0,0,0,0,0,1), ncol=length(inits),
 byrow=TRUE),

  ***# initial covariance of parameters***

                   omega.init = matrix(c(1,0,0,0,0,0,

                                         0,0.1,0,0,0,0,

                                         0,0,0.1,0,0,0,

                                         0,0,0,0.5,0,0,

                                         0,0,0,0,0.1,0,

                                         0,0,0,0,0,0.5), ncol=length(inits), byrow=TRUE),

                   error.model = "constant")

***### fit model***

fit <- saemix(model = mod,

              data = ds,

              control = list(seed=12345,

                             directory="Models/full",

                             save=TRUE,

                             save.graphs=TRUE,

                             print=FALSE,

                             nbiter.saemix = c(750, 250),

                             nbdisplay = 100,

                             displayProgress = TRUE)

              )

***### save fitted model***

saveRDS(fit, "saemix_fit_full.rds")

***########### individual predictions on new data #####################################***

***# load new data***

d_new <- read.csv("data_new.csv")

***### preparations***

***# if saemix package is not loaded: load package***

***# if original data is not loaded: read original data***

***# if model fit is not loaded: read model fit with readRDS() function***

***# if structural model function is not specified, do this (same code as above)***

*(****Box 1****. continued)*

***### initialize new model***

inits <- coefs$fixed  ***# initial values are previous model fit values***

psi <- matrix(inits, ncol=length(inits))

time <- 80

times <- seq(0, time, by = 1)

xidep <- matrix(time, ncol=1)

mod_new <- saemixModel(model = mod_gamma, ***# pass structural model***

                   modeltype = "structural",

                   description = "Gamma model – updated for prediction",

***# same code as before***

                   psi0 = matrix(inits, ncol=length(inits), byrow=TRUE,

                   dimnames=list(NULL,

                      c("amplitude","alpha","beta","base_b0","base_b1","dT")

                      )

                  ),

                   transform.par = c(1,1,1,1,0,1),

                   covariate.model = matrix(c(1,0,0,1,0,0,

                                              1,0,0,1,1,1,

                                              0,0,0,0,0,0,

                                              0,0,1,1,0,0,

                                              0,0,0,0,0,0), ncol=length(inits),
 byrow=TRUE),

***# Only the first parameter is freely re-estimated***

                   fixed.estim = c(1,0,0,0,0,0),

***# same code as before***

                   covariance.model = matrix(c(1,0,0,1,0,0,

                                               0,1,0,0,0,0,

                                               0,0,1,0,0,0,

                                               1,0,0,1,1,0,

                                               0,0,0,1,1,0,

                                               0,0,0,0,0,1), ncol=length(inits),
 byrow=TRUE),

                   omega.init = matrix(c(1,0,0,0,0,0,

                                         0,0.1,0,0,0,0,

                                         0,0,0.1,0,0,0,

                                         0,0,0,0.5,0,0,

                                         0,0,0,0,0.1,0,

                                         0,0,0,0,0,0.5), ncol=length(inits), byrow=TRUE),

                   error.model = "constant")

***### Set intial values to previous estimates***

modnew@psi0["Pop.CondInit",] <- fit@results@fixed.psi

modnew@psi0["Cov.CondInit",] <- diag(fit@results@se.cov)

modnew@omega.init <- fit@results@omega

***### run model for predictions on new data***

***# variables for original and new IDs***

IDs_orig <- unique(d_orig$ID)

n_orig <- length(IDs_orig)

IDs_new <- unique(d_new$ID)

n_new <- length(IDs_new)

*(****Box 1****. continued)*

***# data frames to save predictions***

d_new_pred <- data.frame(matrix(ncol=4,nrow=0))

colnames(d_new_pred) <- c("ID","timepoint", "observed_value", "predicted_value")

d_new_pred_full <- data.frame(matrix(ncol = (time+2), nrow = 0))

colnames(d_new_pred_full) <- c("ID",as.character(unlist(0:time)))

***# loop over participants to refit the model each time***

for (i in 1:n_new){

     ID_new_subject <- IDs_new[i]

     print(paste0("FITTING FOR SUBJECT: ",i,"/",n_new))

     print(paste0("subjectnr: ",ID_new_subject))

***# concatenate original data with new subject***

     d_new_subject <- d_new[d_new$ID == ID_new_subject,]

     ID_new_new <- 1000000 + i ***# give different subject number than train nrs.***

     d_new_subject$ID <- rep(ID_new_new)

     d <- rbind(d, d_new_subject)

***# make saemix data***

     ds <-   saemixData(d,

               name.group=c("ID"),

               name.predictors=c("Cortisol_timepoint"),

               name.response=c("Cortisol_value"),

               name.covariates=c("gender","dummy_1","dummy_2","dummy_3","dummy_4"),

               units=list(x="min", y="nM", covariates=c("-","-","-","-","-","-")),

               name.X="Cortisol_timepoint"

               )

***# update model fit***

     fitnew <- saemix(model = modnew,

               data = ds,

               control = list(seed=12345,

                              directory="gamma_fit",

                              save=FALSE,

                              save.graphs=FALSE))

***# interim save observations with predictions***

     index_obs <- which(fitnew@data@data$ID == ID_new_new)

     y_model <- fitnew@results@ipred[index_obs]

new_pred <- cbind(ID_new_subject,d_new_subject$Cortisol_timepoint,

d_new_subject$Cortisol_value,y_model)

     colnames(new_pred) <- c("ID","timepoint", "observed_value", "predicted_value")

     d_new_pred <- rbind(d_new_pred, new_pred)

***# interim save full model predictions***

     coefsnew <- coef.SaemixObject(fitnew)

     index_subject <- which(attr(ds@nind.obs, "names")  == ID_new_new)

     psi <- t(matrix(unlist(coefsnew$individual$psi$map[index_subject,])))

     xidep <- matrix(0:time, ncol=1)

     d_new_pred_full[i,] <- NA

     d_new_pred_full$ID[i] <- ID_new_subject

     d_new_pred_full[i,2:(time+2)] <- mod_gamma(psi, 1, xidep)

}

***# save predictions***

write.csv(d_new_pred, "Data_new_MM_preds.csv", row.names=F)

write.csv(d_new_pred_full, "Data/Data_new_MM_preds_full.csv", row.names=F)

**References**

Comets, E., Lavenu, A., & Lavielle, M. (2017). Parameter estimation in nonlinear mixed effect models using saemix, an R implementation of the SAEM algorithm. *Journal of Statistical Software*, *80*(3), 1–41. https://doi.org/doi:10.18637/jss.v080.i03

Khoury, J. E., Gonzalez, A., Levitan, R. D., Pruessner, J. C., Chopra, K., Basile, V. S., Masellis, M., Goodwill, A., & Atkinson, L. (2015). Summary cortisol reactivity indicators: Interrelations and meaning. *Neurobiology of Stress*, *2*, 34–43. https://doi.org/10.1016/j.ynstr.2015.04.002

Miller, R., Wojtyniak, J. G., Weckesser, L. J., Alexander, N. C., Engert, V., & Lehr, T. (2018). How to disentangle psychobiological stress reactivity and recovery: A comparison of model-based and non-compartmental analyses of cortisol concentrations. *Psychoneuroendocrinology*, *90*, 194–210. https://doi.org/10.1016/j.psyneuen.2017.12.019

Pruessner, J. C., Kirschbaum, C., Meinlschmid, G., & Hellhammer, D. H. (2003). Two formulas for computation of the area under the curve represent measures of total hormone concentration versus time-dependent change. *Psychoneuroendocrinology*, *28*(7), 916–931. https://doi.org/10.1016/S0306-4530(02)00108-7
